# Dual functionality of tungsten oxide-doped magnesium oxide nanoparticles for organic dye adsorption and antimicrobial activity

**DOI:** 10.1039/d6ra02662a

**Published:** 2026-05-26

**Authors:** Amina Toumi, Habiba A. Hossni, Shahira H. EL-Moslamy, Heba Y. Zahran, V. Ganesh, Mohammad Abohassan, Saleh M. Matar, Elbadawy A. Kamoun, Ibrahim S. Yahia

**Affiliations:** a Department of Health and Laboratory Sciences, College of Medical and Health Sciences, Liwa University P.O. Box 41009 Abu Dhabi United Arab Emirates; b Nanotechnology Section, Egyptian Company for Carbon Materials El-Sheraton/El-Nozha Cairo 11757 Egypt; c Bioprocess Development Department (BID), Genetic Engineering and Biotechnology Research Institute (GEBRI), City of Scientific Research and Technological Applications (SRTA-City) New Borg El-Arab City Alexandria 21934 Egypt; d Laboratory of Nano-Smart Materials for Science and Technology (LNSMST), Department of Physics, Faculty of Science, King Khalid University P.O. Box 9004 Abha Saudi Arabia dr_isyahia@yahoo.com; e Department of Clinical Laboratory Sciences, College of Applied Medical Sciences, King Khalid University Abha Saudi Arabia; f Department of Chemical Engineering, College of Engineering and Computer Sciences, Jazan University Jazan Saudi Arabia; g Department of Chemistry, College of Science, King Faisal University Al-Ahsa 31982 Saudi Arabia ekamoun@kfu.edu.sa badawykamoun@yahoo.com +966 201283320302

## Abstract

The basic fuchsin dye is well known for its long-term stability, potential ecological toxicity in aquatic environments, and genotoxic, neurotoxic, and carcinogenic effects in humans. This study introduces novel tungsten oxide-doped MgO (TW) adsorbent NPs fabricated by applying the sol–gel/auto-combustion method. TW nanoparticles were analyzed using XRD, confirming their cubic structure. Functional groups were examined and verified by FTIR spectroscopy. SEM investigation revealed the quasi-spherical surface morphology of the prepared TW. The optical properties of pure MgO TW0 (4.66 eV) and 10% tungsten oxide-doped MgO TW4 (4.15 eV) were determined using ultraviolet diffuse reflectance spectroscopy (UV-DRS), and BET surface area analysis and electrochemical impedance spectroscopy (EIS) were performed. The effects of variables, such as dose, pH, initial concentration, and contact time on adsorption, were studied, with strong correlation coefficients. TW4 NPs demonstrated great selectivity for BF, providing a maximum capacity of 324.64 mg g^−1^ at pH 7 in BF dye (0.04 g L^−1^) for 35 minutes. The Freundlich adsorption isotherm closely matched the experimental data. Kinetic modeling was studied for BF adsorption using both pseudo-first-order and pseudo-second-order models. The antimicrobial activity of TW NPs was assessed against various human pathogens, including Gram-positive and Gram-negative bacteria and fungal cells. Overall, TW NPs exhibited excellent antimicrobial activity against all tested Gram-negative bacteria, demonstrating their potential as promising nanomaterials for various environmental and medical applications.

## Abstract 2

Dual functionality of the prepared tungsten oxide-doped MgO NPs.

## Graphical abstract

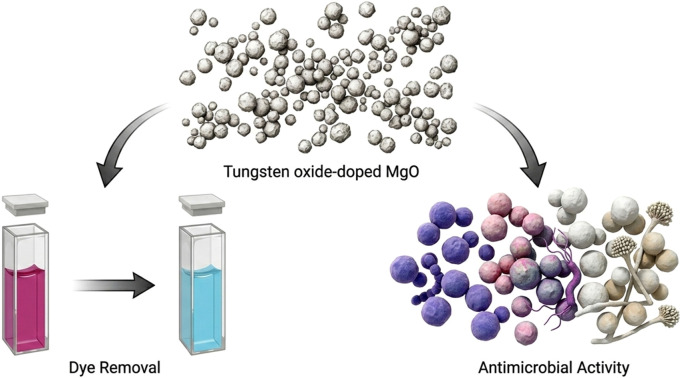

## 1. Introduction

The growth of dangerous environmental contaminants has increased due to human activity and the Industrial Revolution, especially water pollution with excessive levels of organic dyes, posing the most significant ecological problems,^1^ thereby making it difficult to maintain freshwater demand and degrading its physical characteristics. Due to their complex chemical structures, color contaminants used in many industries, *e.g.* the textile, paper, printing, plastic, leather, and cosmetics sectors, are present in many water sources and have significantly negatively influenced the environment.^2^ Basic fuchsin (BF) is a cationic dye known as basic violet or rosaniline chloride, which is used in the textile, leather, paper, and synthetic fiber industries for biological coloring. It has anesthetic, antibacterial, fungicidal, and inflammable properties but is also hazardous due to its high toxicity, weak biodegradability, and suspected carcinogenicity. Its exposure can cause eye burns, nausea, vomiting, diarrhea, and skin and respiratory irritation. Concerns have grown due to its long-term impact on the environment and human health.^3–6^ Because of dye's low biodegradability and environmental risks, dye pollutants, and cancer treatment using the currently known procedures typically lead to immunosuppression in patients, putting them at a high risk of serious microbial infections. It is critical to develop novel anticancer drugs with integrated antimicrobial activity.^5^ Surface-functionalized NPs are now broadly utilized in a diversity of disciplines, *e.g.* environmental science and medicine. Antimicrobial treatments, such as antibiotics and surface modifications of hospital facilities and equipment, have been developed to reduce the prevalence of nosocomial infections. However, antibiotic resistance has rendered most of them ineffective and unsustainable. As a result, research on alternative materials capable of combating pathogenic microbes has been continued.^7^ Recently, new potential uses of nanomaterials and nanocomposites have emerged to fight against antimicrobial resistance in order to reduce the incidence of these pathogens.^8^ Due to the importance of dye removal from wastewater, reusable and highly selective nanomaterials with rapid performance and simple methods for removing dyes from industrial wastewater have become a focal point for environmental researchers, with removal efficiencies approaching 99.9%.^9,10^ Several methods have been used to remove these dangerous dyes from wastewater.^11^ Adsorption is a popular, efficient, and economical method for treating water owing to its ease, efficiency, and convenience, making it a promising method for water treatment compared with other color removal techniques.^12^

Several studies have been conducted on the removal of the basic fuchsin dye, including that of Ba Mohammed *et al.*, who synthesized Fe-ZSM-5 zeolite for BF adsorption in an aqueous solution.^13^ Zhang *et al.* use Fe_3_O_4_@HA nanocatalysts that serve a dual environmental adsorption purpose.^14^ Moreover, MgO/BaCO_3_/BaCrO_4_/C nanocomposites were functionalized as adsorbents to remove the basic fuchsin dye from their solutions.^15^ As a result, research on removal processes frequently uses this dye as a model pollutant.

In recent years, interest has been increasing in the fabrication of metal oxide NPs,^16^ particularly magnesium oxide (MgO) NPs, owing to their distinct advantages. These include being environmentally friendly,^17^ readily available,^18^ and possessing notable physical, chemical, and optical properties.^19^ MgO has found applications, including in paints, pharmaceuticals, and water purification, as a catalyst.^20–22^ For the treatment of wastewater contaminated with dyes, they are synthesized by various techniques to produce metal oxide nanoparticles, including hydrothermal, pyrolysis, sol–gel, microwave irradiation, laser vaporization, and wet chemical methods.^23–27^ The sol–gel method provides outstanding control over the purity, morphology, and particle size at comparatively modest processing temperatures.^28^ When working with magnesium oxide nanoparticles, factors such as homogeneity, surface properties, low energy consumption, and the absence of expensive or complex equipment are important, and this degree of precision is critical because of their large surface area and distinctive physicochemical characteristics. Nanomaterials, including metals and metal oxides, are useful for dye removal and antibacterial applications.^20,29^ Previous studies have successfully demonstrated the synthesis of uniform MgO nanoparticles using the sol–gel process and highlighted its effectiveness in manufacturing high-purity materials with consistent particle sizes, providing support for this decision.^30^ MgO is a mineral that is mostly applied in medicine to treat numerous diseases, including heartburn and stomach pain. MgO NPs may have prolonged antimicrobial effects due to their low volatility and excellent temperature stability.^9,31^ Besides, MgO NPs fabricated using the sol–gel method have shown exceptional efficacy in eliminating dye pollutants from aqueous solutions.^28^ Their research demonstrated that, under ideal conditions, the nanoparticles had a high adsorption capacity, highlighting the promise of sol–gel-produced magnesium oxide as a viable, environmentally benign treatment option for wastewater containing organic colors.^32^ Moreover, metal oxide nanoparticles (ZnO, SnO_2_, WO_3_, MgO, TiO_2_, *etc.*) are functionalized in various sectors, such as biology, electrochemistry, environmental science, and medicine.^3,7^ They have a significant surface area-to-volume ratio, are optical and magnetic, and have a high surface energy compared to their bulk counterparts.^8,10^ Some well-known nanoparticles (NPs) (Ag, Cu, Ti, and others) have limited economic applicability due to their high biotoxicity. To address the toxicity issue, researchers have investigated oxide NP as a more effective option. Moreover, the surface modification of NPs has been proposed to broaden the applicability range of NPs while lowering their side toxicity.^12,33^ Among its promising uses, the removal of synthetic dyes in the researchers' area has made significant progress.^34–37^ Tungsten oxide significantly improves the catalytic activity of metal oxides (*e.g.* ZrO_2_) by modifying the oxidation states and surface structures of the active sites.^38,39^ Moreover, the addition of the WO_3_ catalyst achieved a capacity rate of 78.14 mg g^−1^ and maintained its performance throughout the adsorption period, greatly enhancing the removal activity and stability.^40^

Herein, the main aim of this study is to use tungsten oxide-doped MgO NPs as adsorbent NPs for the basic fuchsin dye from an aqueous solution to address multipollutant removal while maintaining the antimicrobial activity of NPs. The contact time, pH, dosage, and dye concentration were studied and discussed. To understand the equilibrium and adsorption mechanism, kinetic and isothermal investigations were conducted and discussed in detail. Moreover, the antimicrobial activity of the prepared tungsten oxide-doped MgO NPs was tested against Gram +ve bacteria (*Staphylococcus aureus* and *Bacillus cereus*), Gram −ve bacteria (*Klebsiella pneumoniae*, *E. coli*, *Klebsiella rhinoscleromatis*, and *Salmonella* organisms), and fungal cells (*Candida glabrata* and *Candida albicans*).

## 2. Materials and methods

### 2.1. Materials

**Fig. 1:**
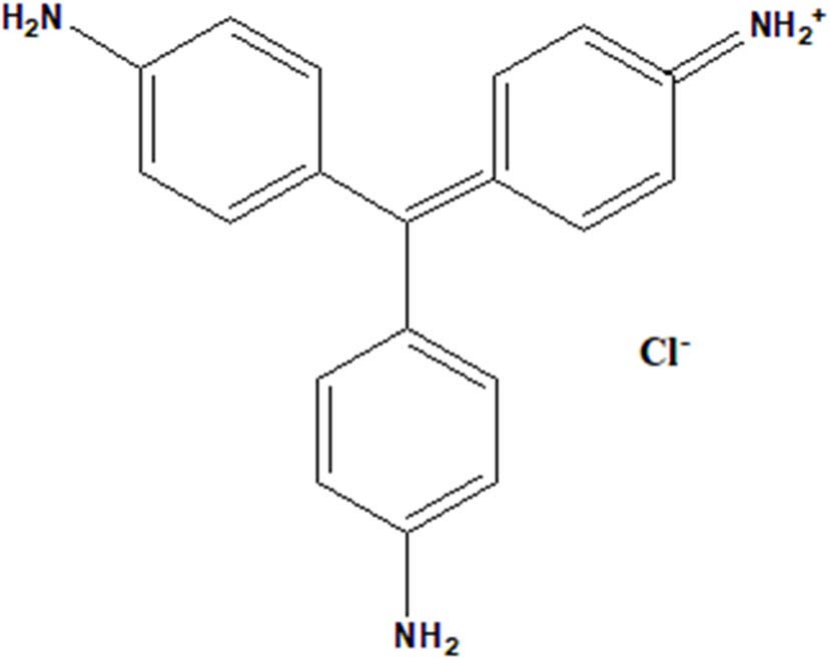
Chemical structure of the basic fuchsin micro-stain dye.

Magnesium nitrate hexahydrate (Mg(NO_3_)_2_·6H_2_O, 99%) was used as the precursor of magnesium. Sodium tungstate dihydrate (Na_2_WO_4_·2H_2_O, 98%), sodium chloride (NaCl, 98%), and sodium nitrate (NaNO_3_, 98%) were obtained from Loba Chemie, India. Tannic acid (C_76_H_52_O_46_) was obtained from Research Labin, India. Ascorbic acid (C_6_H_8_O_6_) was obtained from Sigma-Aldrich, Germany). Hydrochloric acid (HCl) and isopropyl alcohol (C_3_H_8_O, 99%) were bought from Alpha Aeser, India. Sodium hydroxide (NaOH) was purchased from Merck, Germany. The organic dye, *e.g.* fuchsin basic micro-stain, was obtained from Loba Chemie, India (C_19_H_17_N_3_HCl, as shown in Fig. 1, and double-distilled water was applied to every step of the production process. The antimicrobial activity of WO_3_/MgO NPs was tested using some human pathogens, *e.g.* Gram +ve bacteria such as *Staphylococcus aureus*, *Bacillus cereus* and fungal cells like *Candida glabrata* and *Candida albicans*. In addition, Gram −ve bacteria such as *Klebsiella pneumoniae*, *E. coli*, *Klebsiella rhinoscleromatis*, and *Salmonella* organisms were screened. These human pathogens were helpfully obtained from GEBRI, SRTA-City, Alexandria, Egypt.

### 2.2. Preparation of TW NPs

**Fig. 2:**
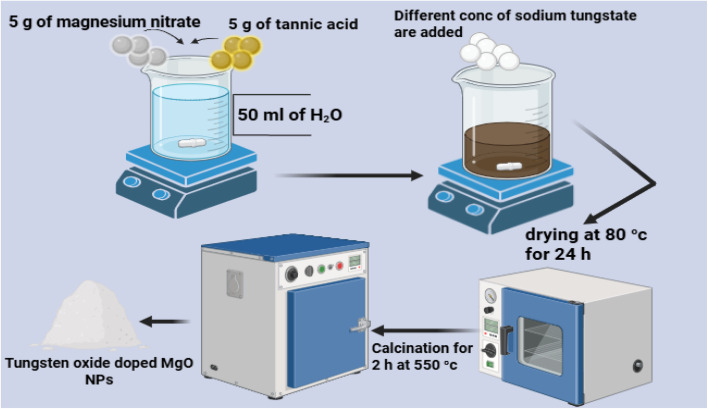
Schematic of the preparation of the WO_3_-doped MgO NPs using the sol–gel/auto-combustion method.

Tungsten oxide-doped magnesium oxide (TW) NPs were synthesized by adding 5 g of Mg(NO_3_)_2_·6H_2_O as a precursor to 50 mL of DDW containing 5 g of tannic acid. The mixture was continuously stirred for one hour at room temperature. After dissolution, different concentrations of sodium tungstate (0%, 1%, 3%, 5%, 10% and 30%, w/v) were added as a doping material. After a further 30 min of stirring, the solution was dried at 80 °C for 24 h.^41^ Subsequently, the obtained NPs were calcined at 550 °C for 2 h (Fig. 2). Finally, all the fabricated NPs were designed, as listed in Table 1, and fully characterized at the Egyptian Company for Carbon Materials (ECCM), Cairo.

**Table 1:** Symbol definition for the tungsten oxide-doped magnesium oxide NPs

Name	Sample code
WO–MgO	TW
Pure MgO	TW0
1% WO–MgO	TW1
3% WO–MgO	TW2
5% WO–MgO	TW3
10% WO–MgO	TW4
30% WO–MgO	TW5

### 2.3. Adsorption batch of the basic fuchsin (BF) micro-stain dye

A batch of adsorption trials was accomplished for dye adsorption to estimate the adsorption of the basic fuchsin (BF) micro-stain dye onto the TW NP surface. 0.01 g catalyst NPs were loaded onto a 10 ppm BF stock solution in a 100 mL volume. The starting dye concentration was increased from 2 to 40 ppm, while the NP dosage varied from 0.002 to 0.02 g, the pH was adjusted from 4 to 9, and the reaction time extended from 5 to 35 minutes at room temperature on a magnetic stirrer with continuous stirring of 450 rpm to conduct the adsorption experiments. 0.1 mol per L HCl and 0.1 mol per L NaOH solutions were used to keep the pH of the solution at the desired level. Flasks were taken out every 5 min, and a UV-vis spectrophotometer calibrated to the maximum absorption wavelength was used to determine the amount of unabsorbed basic fuchsin micro-stain. The various adsorption factors (contact time, pH, dosage, and initial concentration) affecting TW adsorption efficiency were studied using eqn (1)^42^ as follows:1
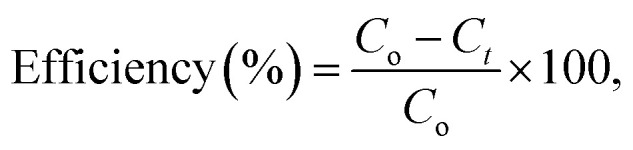
where *C*_o_ and *C*_*t*_ are the initial and residual BF absorbance, respectively.

### 2.4. Antimicrobial activity

The antimicrobial potential of TW NPs against various human pathogens was evaluated using the agar-well diffusion method.^43^ In brief, tested bacterial pathogens were cultivated at 37 °C ± 1.5 °C in Luria-Bertani (0.5% yeast extract, 1% NaCl, and 1% tryptone) medium, while fungal cells were grown at 28 °C ± 2.5 °C in nutrient broth medium (0.1% beef extract, 0.2% yeast extract, 0.5% peptone, and 0.5% NaCl) for 18 h. After that, the turbidity of the cultures was altered to a freshly prepared 0.5 McFarland turbidity standard (10^8^ CFU mL^−1^) with fresh broth media.^44^ Then, each pathogen is swabbed homogeneously onto separate agar plates using sterile swabs under sterile conditions. The wells are then created using a sterile polystyrene tip (5 mm). An ultrasonic cleaner was used to prepare and homogenize various TW NP suspensions (10, 30, 50, 70, 90, and 110 µg mL^−1^). After that, 50 µL of each TW NP suspension was loaded into each well. After each plate was incubated at 37 °C for 72 h, inhibition zones around each well were measured in millimeters (mm) to calculate the lowest concentration that inhibits microbial growth (MIC). To estimate their relative inhibitory potency (%), 48 mL of freshly prepared bacterial cultures (OD_600_ ≈ 0.3) were treated with 2 mL of each TW NP concentration (30, 50, and 70 µg mL^−1^) for 48 h.^45^ At the same time, a control group (cultures that had not been treated) was prepared. Then, these tubes were inoculated in a rotary shaker (200 rpm) at 37 °C. A UV-visible spectrophotometer was used to detect the optical density of withdrawn samples at 600 nm every 6 h. Relative inhibitory potency of TW NPs against human pathogens (%) was calculated using the following formula:^43^2
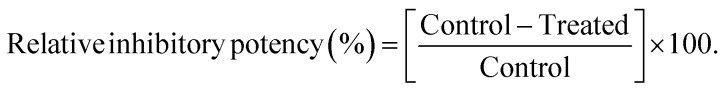


Additionally, the bacterial cells were treated with 300, 250, 200, 150, and 100 µg mL^−1^ of TW NPs to determine the minimum lethal concentration (MLC), which is the lowest antimicrobial dose required to kill 99.9% of tested pathogens.^46^ All these assays were performed in triplicate. All of the attempts were made in triplicate, and the data were recorded as mean ± standard deviation.

### 2.5. Characterization

XRD (Shimadzu LabX-XRD-6000, Japan) was used to analyze the structure using Cu Kα1 radiation (*λ* = 1.54 Å) and 2*θ* ≈ 10°–80°. The X'pert High Score software was utilized to index the XRD peaks. Functional groups at the *ν* 4000–400 cm^−1^ range were detected using an FT-IR spectrophotometer (Nicolet 6700, Thermo Fisher Scientific, Waltham, MA). Using SEM-EDX, morphology and elemental content were investigated. Following gold (Au) coating, a (JASCO V-650) double-beam spectrophotometer with a photomultiplier tube detector and single monochromatic beam was used to capture UV-vis/DRS spectra. The Brunauer–Emmett–Teller (BET) surface area of the sample was determined using an N_2_ adsorption/desorption analysis of the TW NPs at 77 K (BELSORP-miniX 10,115, Japan). Electrochemical impedance spectroscopy (EIS) and other electrochemical measurements of the electrolyte were established using an electrochemical workstation (CHI6005E).

## 3. Results and discussion

### 3.1. XRD analysis

**Fig. 3:**
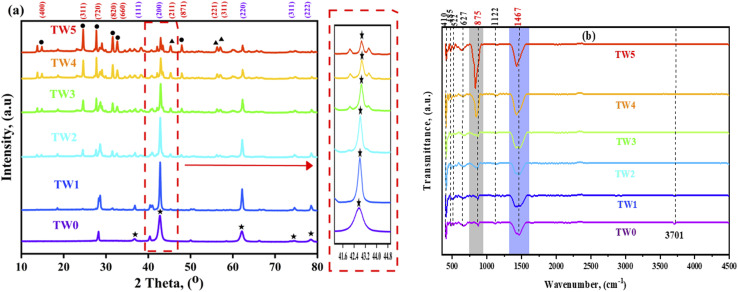
(a) XRD patterns of the TW0 and TW NPs. (b) FTIR spectra of the TW NPs.

Fig. 3a illustrates the XRD diffraction of pure MgO (TW0) and tungsten oxide-doped MgO (TW) NPs with different doping ratios (1%, 3%, 5%, 10%, and 30%, w/v) (Table 1). Diffraction peaks were well indexed to the cubic crystal structure (card no 00-045-0946 and 04-012-6481). The strong predominant MgO diffraction peaks were visible in all the patterns. This is an indication of the formation of MgO NPs, which is supported by findings from reported literature values.^47,48^ The change in dopant concentration leads to the continuous transformation of peak intensity after doping. XRD patterns show that tungsten oxide nanoparticles have a mixed phase, tetragonal and hexagonal crystal structure at the highest doping concentration according to (card no. 01-075-2187 and 00-041-0745). Fig. 3a demonstrates that the intensities of the peaks of TW nanoparticles decreased and shifted to lower 2*θ* values. Doping ions cannot be included in the MgO host lattice sites of NPs, which undergo tungsten oxide ion oxidation to segregate at the grain boundaries and on the grain of MgO nanoparticles.^49^ The *Scherer* formula was applied to calculate crystallite size (*D*), dislocation (*δ*), and lattice strain for nanocatalysts through the given eqn (3)–(5)^50^ as follows:3
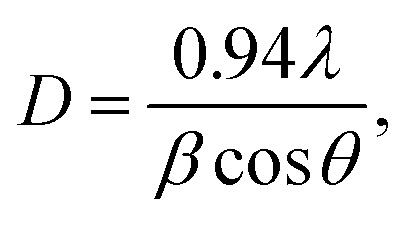
where *λ* is the wavelength of X-ray radiation and *β* is the full width at half maximum (FWHM) of peaks at diffraction angle 2*θ*. The study found that the size of nanocrystals increased with increased concentrations of tungsten oxide, with sharper crystallite peaks and an average crystallite size. All the results of the average crystallite size for doping concentration are shown in Table 2 as follows:4
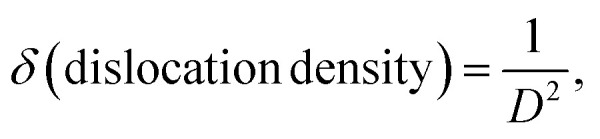
5
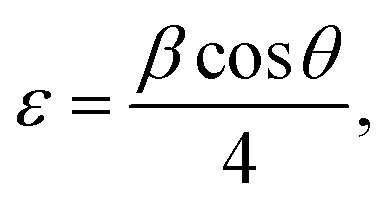
where *β* is the full width at the half maximum (FWHM) and *λ* is the wavelength, which was determined in Fig. 3a. The results obtained are condensed in Table 2. According to the data, the crystalline size began to increase as the doping concentration increased, and the lattice strain gradually increased. This behavior shows that the dopant and host ions have different ionic radii and charges, causing localized lattice distortions that raise the internal strain in the crystal structure.^51,52^

**Table 2:** X-ray diffraction data of the TW NPs

Catalyst	Mean grain size (nm)	Mean dislocation (*δ*)	Mean lattice strain (*ε*)	Direct band gap (eV)
TW0	24.15869	2.586 × 10^−3^	1.668 × 10^−3^	4.66
TW1	71.4251	2.427 × 10^−4^	5.234 × 10^−4^	4.26
TW2	60.98414	3.196 × 10^−4^	6.043 × 10^−4^	4.18
TW3	62.94945	2.641 × 10^−4^	5.592 × 10^−4^	4.06
TW4	125.3868	2.005 × 10^−4^	5.474 × 10^−4^	4.15
TW5	174.7556	2.400 × 10^−4^	5.350 × 10^−4^	4.21

### 3.2. FTIR analysis

FTIR spectroscopy was used to study the TW chemical structure, bonding, and functional groups. Fig. 3b shows the bonds and functional groups within the *ν* 4000–400 cm^−1^ range. Absorption peaks at *ν* 3701, 1467, 1122, 875, 627, 522, 485, and 410 cm^−1^ are identified in the TW nanocomposite and TW0 spectra. A small peak indicates the O–H stretching mode at *ν* 3701 cm^−1^. The bending vibration of H_2_O molecules is recognized as the cause of the peak at *ν* 1467 cm^−1^. The FTIR spectrum presents peaks at the *ν* 800–600 cm^−1^ range, linking to several W–O bonding vibrations in tungsten oxide, such as O–W–O and W–O–W stretching and bending modes.^53^ Mg–O vibrations were confirmed to be present by the primary peaks at *ν* 485 and 410 cm^−1^.^54^ As observed, the disappearance of the hydroxyl group at *ν* 3701 cm^−1^ by increasing the doping concentration is because of the binding of tungsten oxide to MgO NPs.^55^

Moreover, sharper bands provided a more ordered surface structure of MgO, supporting the narrow crystalline peak in the X-ray data where distinct carbonate groups and Mg–O vibrations develop at *ν* 1467 cm^−1^. This is ascribed to a change in bond length resulting from the doping of tungsten oxide in the MgO crystal lattice, reflecting the presence of TW, which shows a slight change in transmittance intensity, verifying successful doping into the MgO lattice.

### 3.3. SEM, TEM, SAED and HRTEM investigation

**Fig. 4:**
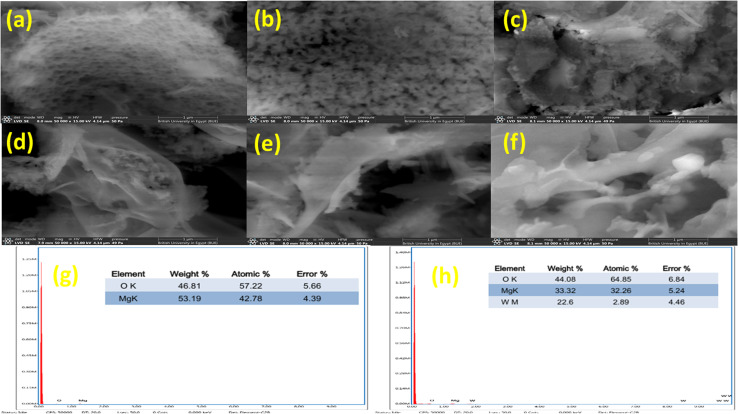
SEM images of the surface morphological properties of (a) TW0 and (b–f) different concentrations of TW. (g and h) EDX analysis of TW0 and TW4.

SEM analysis was performed to study the morphology and to determine the grain sizes of TW0 and TW at different doping concentrations. SEM images of TW in Fig. 4a–f show particle formation with regular and well-defined quasi-spherical morphology.^56^ TW1 exhibits larger particle formation than that obtained for TW0 (Fig. 4a and b). The change in the morphology and grain size of TW (TW2, TW3, TW4, and TW5) particles is related to the doping ion inclusion. The contact zone between the particles is missing due to the agglomeration of the particles. Therefore, an increase in doping concentration impacts grain size. As shown in the XRD data. Fig. 4g and h demonstrates the EDX analysis carried out to identify the purity and chemical composition of TW0 and TW4. Mg and O are the most common elements in the structure, with weight percentages of 53.19% and 46.8%, as shown in Fig. 4g, while W also found in tungsten oxide-doped MgO confirms the presence of Mg, O, and W, with 33.42%, 44.08%, and 22.6% (Fig. 4h), The uniform distribution of elements indicates that WO and MgO phases were well mixed and in close contact inside the nanostructure.

**Fig. 5:**
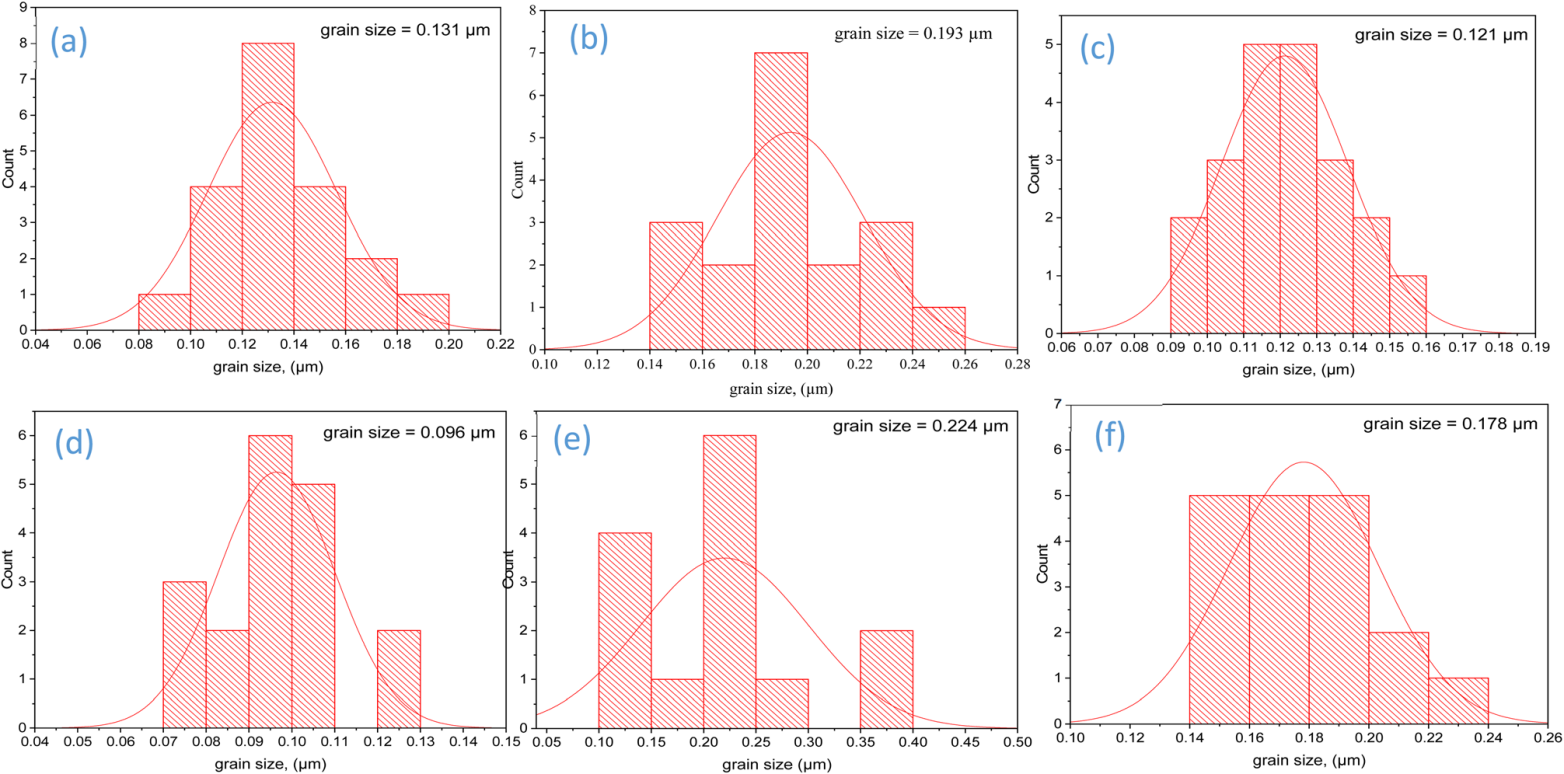
Size-distribution histograms of (a) TW0 (a) and (b–f) different concentrations of TW NPs.

**Fig. 6:**
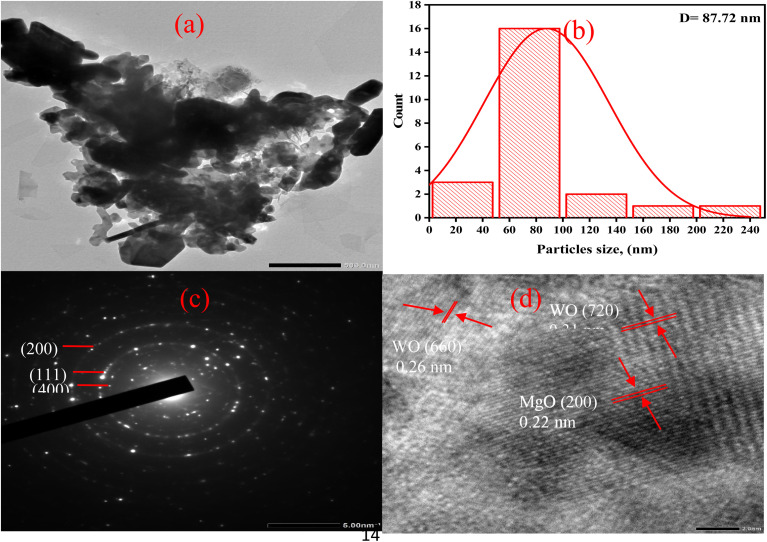
(a) TEM image of TW4. (b) Size-distribution histogram of TW4. (c) SAED pattern and (d) HRTEM image of TW4.

Fig. 5 presents the TW0 and TW size distribution histograms. The sizes of the grains were determined using the ImageJ software. The determined size was the grain length for TW0 and TW at different concentrations, as shown in Fig. 5a–f. The tungsten oxide combination in the magnesium oxide crystalline structure leads to a larger grain size than in TW0 owing to the agglomeration of particles. The grain size varies from 0.096 µm at TW0 to 0.224 µm at TW4.^57^ Additionally, the quasi-spherical form of TW4 is confirmed by a TEM picture (Fig. 6a), illustrating a quasi-spherical structure with a particle size of 87 nm, as shown in Fig. 6b. The SAED pattern of the TW4 composite shows the coexistence of two distinct crystalline systems with reciprocal space for crystallographic interpretations of the generated sample. Fig. 6c displays a high-resolution transmission electron microscopy (HR-TEM) picture of a single nanoparticle. The illustration clearly shows that the nanoparticle is highly crystalline, with distinct lattice fringes visible throughout. XRD analysis results and observed crystallographic “*d*” values are in good agreement.

### 3.4. UV-visible spectroscopy

**Fig. 7:**
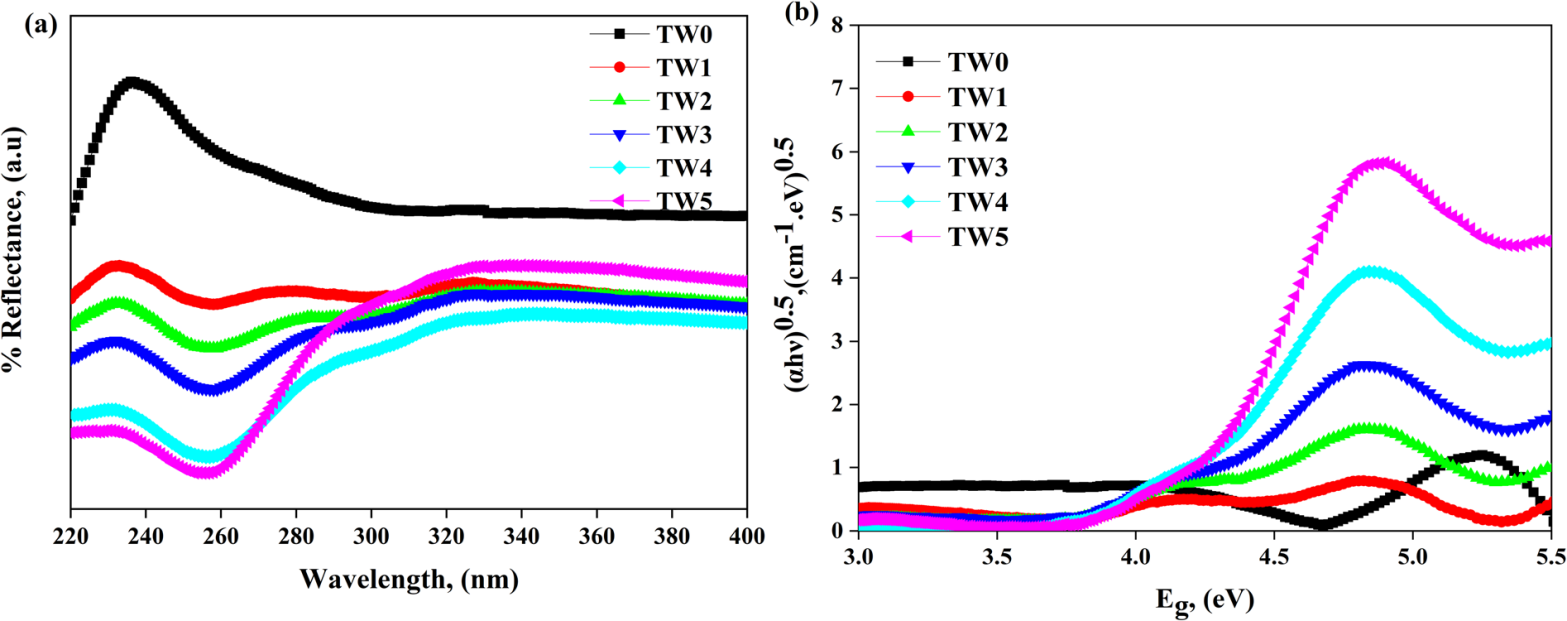
(a) UV-vis DRS spectra of the TW NPs and (b) direct allowed bandgaps.

The optical properties of TW0 and TW NPs were studied using UV-vis. spectroscopy. Indicating band-to-band electrical transition from valence band to conduction band, UV-vis. absorption spectrum shows a notable absorption peak at *λ* = 236 nm (Fig. 7a). This distinct peak indicates the effective production of MgO NPs at the nanoscale and reflects their distinctive optical properties, which are in accordance with previous studies,^58,59^ showing a decrease in reflectivity after the incorporation of MgO with tungsten oxide. To detect the band gap, a Tauc plot is calculated to determine the band gap energy values of the NPs using eqn (6)^60,61^ as follows:

6 (*αhν*) = *A*(*hν* − *E*_g_)^*n*^,

where *α* is the optical absorption coefficient, *hν* = photon energy, *E*_g_ is the direct band gap, *A* = constant, 3*n* = 1/2 for direct allowed transitions, and *n* = 2 for indirect allowed transitions. Fig. 7(a) and (b) show the Tauc plot, where the linear portion of the curve is extrapolated to intercept the photon energy *hν* axis at (*αhν*)^0.5^. TW shows a lower band gap than TW0, calculated optical band gap energy from 4.66 eV to 4.06 eV (Table 2), which is consistent with previous studies.^52,62^Fig. 7 illustrates how doping resulted in a notable alteration in the band structure, reflecting the material's considerable modifications and the improvement towards a reduced band gap.^58^

### 3.5. BET analysis

**Fig. 8:**
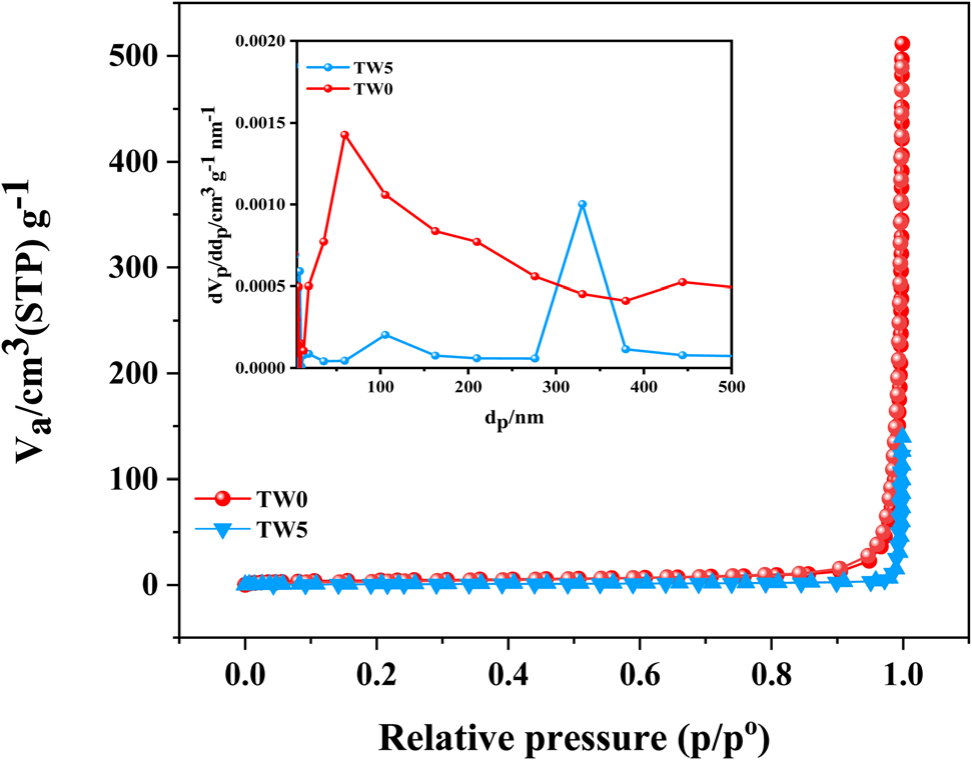
N_2_ adsorption–desorption isotherms and pore size distribution curves of TW0 and TW5.

N_2_ adsorption–desorption curves for TW0 and TW5 are shown in Fig. 8. The appropriate properties for these materials, including surface area, pore diameter, and pore volume, are illustrated in Table 3. Microporosity is evident from the isotherm's characteristic Type IV pattern with a noticeable hysteresis loop (Fig. 8). The specific surface areas of TW0 and TW5 NPs are 14.55 and 3.7235 m^2^ g^−1^, respectively, using the BET technique. The microporous nature of the nanoparticles was confirmed using the BJH method, indicating that the pore volumes were found to be 53.98 and 102.72 nm, respectively (Table 3). Although we discovered that doping considerably decreased the surface area, this is not the main cause of enhanced adsorption. Doping may produce oxygen groups and new chemical functionalities on the surface, which greatly aid in attracting molecules even though the material's optical qualities are enhanced.^63^ The primary cause of the materials' porosity is the aggregation of quasi-spherical nanoparticles, which reduces the surface area.^37^

**Table 3:** BET isotherm and BJH pore size distribution analysis of TW0 and the TW NPs

Sample	BET surface area (m^2^ g^−1^)	Pore volume (cm^3^ g^−1^)	Pore diameter (nm)
TW0	14.55	0.41	53.99
TW5	3.72	0.085	102.72

### 3.6. Impedance spectroscopy

**Fig. 9:**
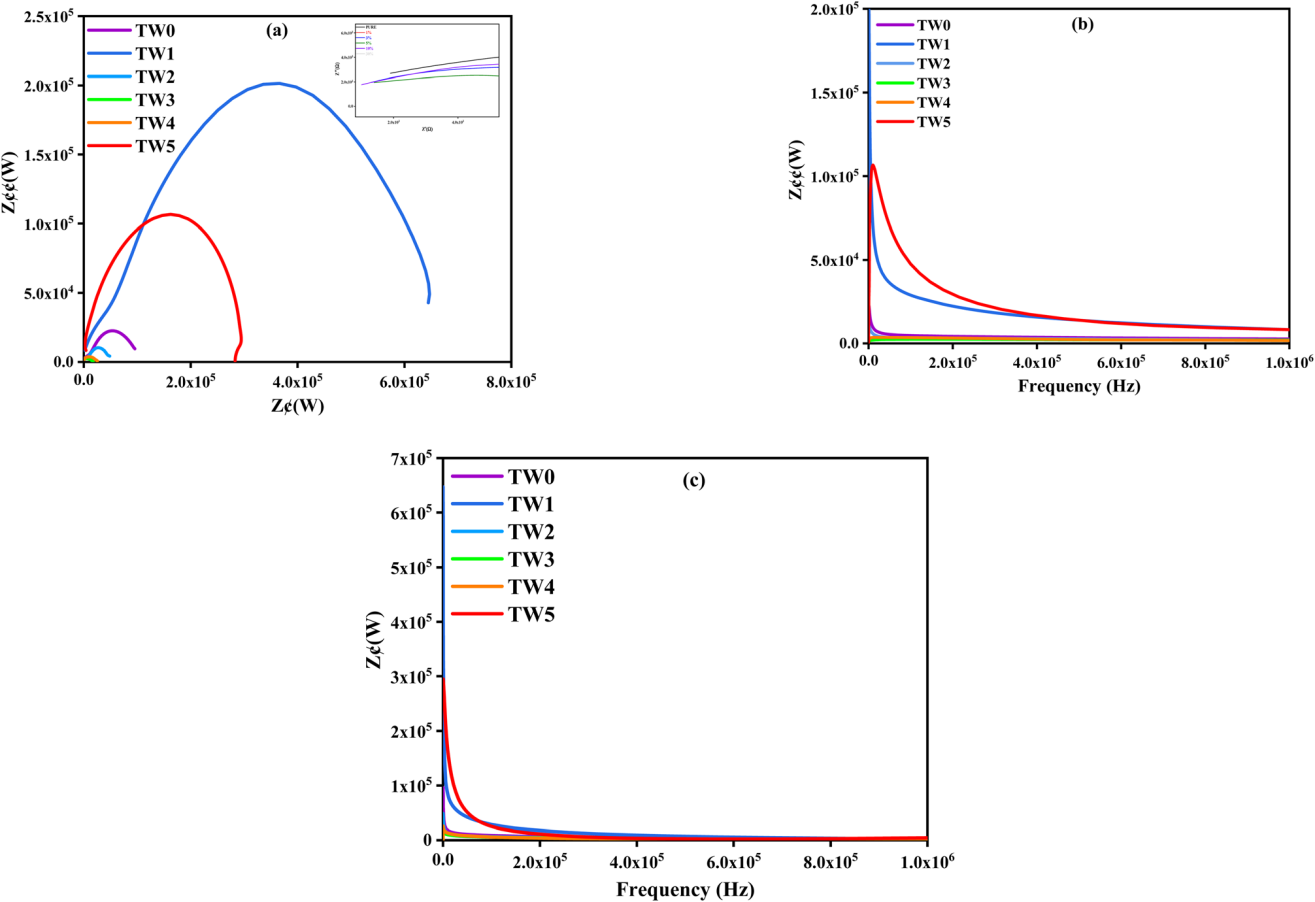
Nyquist plots showing the relationships between (a) (*z*′, *z*″), (b) (frequency, *z*″), and (c) (frequency, *z*′) of TW NPs.

Fig. 9a demonstrates the relation (real (*z*′) *vs.* imaginary impedance (*z*″)) for TW samples, as semicircles at low frequency describe the charge transfer impedance (*R*_ct_) and start with initial impedance (*R*_s_) at high frequency. Sample TW4 exhibits the lowest *R*_s_ at 1008 Ω as the optimum concentration at the optimum range of TW2–TW4, where samples TW2 and TW5 show values of 1306 and 1393 Ω, respectively. Fig. 9(b) and (c) show the relations (frequency *vs.* (*z*″)) and (frequency *vs.* (*z*′)), where samples TW1 and TW5 are outside the optimum range, emphasizing the unlined relation between concentration and conductivity. Sample TW1 mentioned above, with a heterogeneous mixture due to a low concentration, leads to defects in the MgO structure lattice and acts as a block grain boundary for charge carriers, resulting in increased *R*_s_ and *R*_ct_, as well as a significant decrease in the constant phase element (CPE) at the same voltage (5 V) and frequency range (0–10^6^ Hz). In comparison to TW0 and other dopants, especially TW4, we discover that *R*_ct_ gives the lowest value (Table 4), demonstrating enhanced surface charge transfer and dye interaction with the formation of efficient chemical binding sites for BF dye. Additionally, the highest CPE statistics are associated with surface activity, more dye binding sites, robust molecular interaction, and improved dye ion transport.^64,65^

**Table 4:** Fitting parameters of the ZView software

Sample	*R*_s_ × 10^−8^	CPE (µF)	*R*_ct_ × 10^5^
TW0	0.22	1.4	1.08
TW1	15.8	0.34	6.98
TW2	0.08	1.9	0.5
TW3	0.14	1.3	0.19
TW4	0.001	1.88	0.26
TW5	2.34	1.18	3.08

### 3.7. Adsorption performance

TW0 and TW NPs were used as adsorbents in a batch test for the adsorption of BF on the effect of varying doping concentration with contact time. Then, the best samples were tested with various factors, such as pH, amount of adsorbent, and initial concentration of dye, in an aqueous medium varied to minimize material consumption, reduce cost, and ensure environmental efficiency.

#### 3.7.1. Effect of contact time

**Fig. 10:**
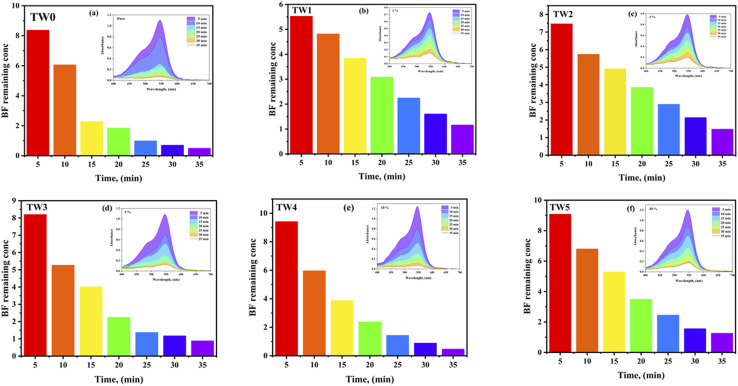
(a–f) Absorption spectra of remaining BF dye concentration, along with the corresponding UV-vis absorption spectra presented as insets within each figure.

**Fig. 11:**
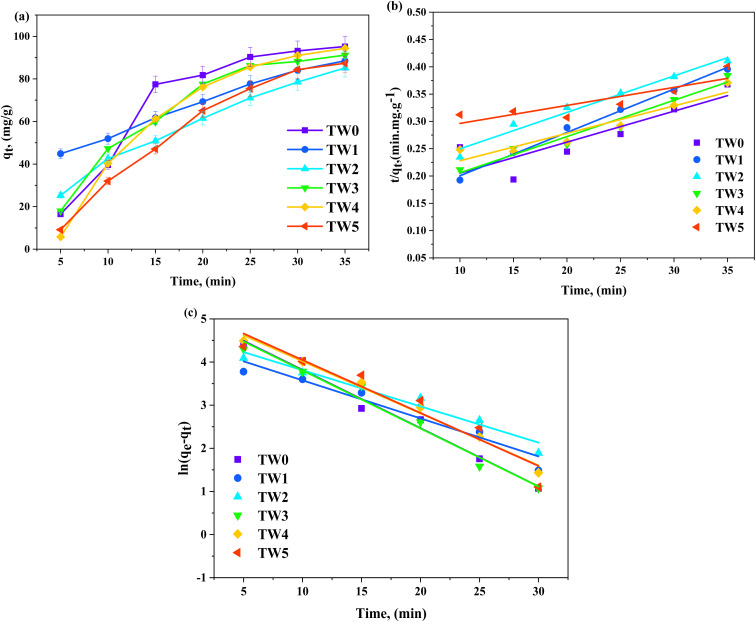
(a) Contact time, (b) PFO, and (c) PSO plots of the TW NPs.

Using pure MgO (TW0) and tungsten oxide doped-MgO (TW) nanoparticles, the effect of contact time interval on BF adsorption was tested from 5 to 35 minutes in 5-minute steps (dye conc: 10 ppm; adsorbent dose: 0.01 g; pH: 7; stirring speed in dark: 450 rpm; under ambient conditions). The study also assessed the effect of the catalyst doping concentration on BF removal. The results are illustrated in Fig. 10. Over time, initial adsorption improves slowly with time until reaching equilibrium, where the curve plateaued around 35 min, indicating a nearly constant level, highlighting the optimal contact time for the removal efficiency of TW0 and TW4 NPs with adsorption capacities of 95.19 mg g^−1^ and 94.41 mg g^−1^, respectively (Fig. 11a). This suggests that the contact duration and doping level affected the adsorption capacities. The process did not continue to increase, indicating a reduction in accessible empty sites that could potentially impact this phenomenon.^66^ As a result, the removal effectiveness of the doped samples was almost identical to that of pure doping, especially at an ideal dopant concentration. Other doping concentrations have a lower removal due to the fewer active adsorption sites accessible for dye molecules, which eventually lowers adsorption effectiveness.^67–69^Eqn (7) is used to calculate the quantity of BF dye adsorbed per gram of the adsorbent (mg g^−1^) as follows:7
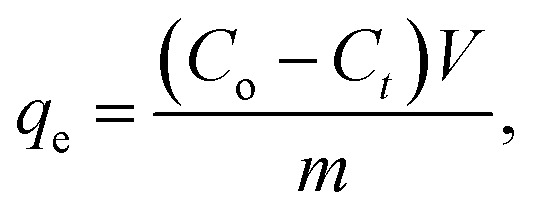
where *C*_o_ is the starting concentrate on (mg L^−1^) of BF dye (mg L^−1^), *C*_*t*_ is the concentration of BF dye at time *t*, m is the mass of the adsorbent (g), and *V* is the volume of the solution (L). To evaluate the adsorption performance of TW for BF dye removal, the equations for the pseudo-first-order (PFO) and pseudo-second-order (PSO) kinetic models are studied, as illustrated in Fig. 11(b and c).^2^Table 5 displays the experimental, computed, and correlation coefficient (*R*^2^) data. It is observed that pure material conforms to the model better, which means that TW0 depends only on the initial concentration and active site due to homogeneity, without the chemical composition on the surface rather than simple physisorption.^70^ With increased doping to TW1 and TW2, it has been observed that it has transformed into a chemisorption state due to the addition of a new network site.^71,72^ However, during TW4 and TW5 doping, the material begins to become saturated with doping, and chemical homogeneity decreases due to agglomeration and a reduction in the surface area.^73^ The results indicate that the superiority of this choice under current conditions does not negate the possibility of achieving an optimal performance level.

**Table 5:** Kinetic models of the TW NPs

Kinetic models	Non-linear equation	Parameters	Pure	1%	3%	5%	10%	30%
Experimental data	—	*q*_e_ (exp)	95.19	88.49	85.17	91.15	94.41	87.36
Pseudo-first order	*q*_*t*_ = *q*_e_(1 − e^−*kt*^)	*K*_2_ (g mg^−1^ min^−1^)	0.13	0.08	0.08	0.13	0.12	0.12
		*q*_e_ (cal)	175.30	86.24	104.44	172.60	185.57	195.23
		*R* ^2^	0.98	0.92	0.95	0.97	0.98	0.92
Pseudo-second order	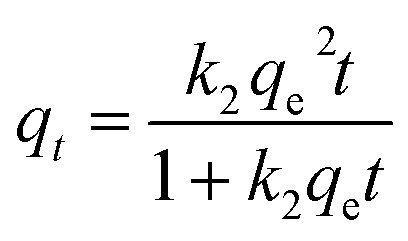	*K* (min^−1^)	2.16 × 10^−4^	5.21 × 10^−4^	2.44 × 10^−4^	3.17 × 10^−4^	1.42 × 10^−4^	4.13 × 10^−5^
		*q*_e_ (cal)	176.366	125.944	149.700	150.37	198.807	303.03
		*R* ^2^	0.74	0.99	0.97	0.96	0.90	0.76

#### 3.7.2. Effect of pH

**Fig. 12:**
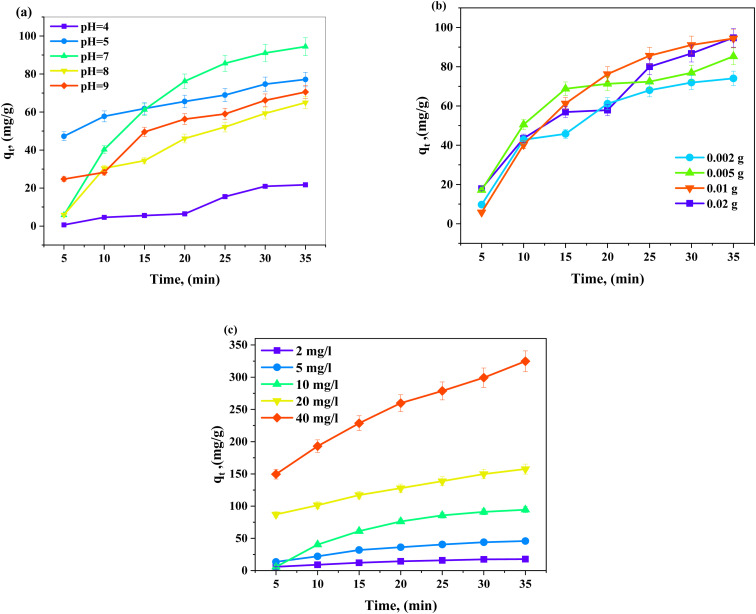
Effect of (a) pH, (b) adsorbent dose, and (c) dye concentration on the adsorption of BF by TW4.

The pH of the solution is adjusted to 4.0–9.0, and the dye pH shows that basic conditions are ideal for BF adsorption onto TW at pH = 7, as shown in Fig. S1 (SI). The adsorption effectiveness was shown to slightly decrease by 70.52 mg g^−1^ at pH = 9; its effectiveness was significantly diminished in an acidic environment. Moreover, a drop was observed at pH = 2, which reached 21.68 mg g^−1^ (Fig. 12a), suggesting that acidic conditions were less conducive to dye adsorption; its effectiveness was significantly diminished in an acidic environment. This is ascribed to the catalyst surface having a more negative charge, which strengthens the interactions with positively charged species and promotes adsorption and catalytic processes. However, there was no discernible difference in performance since the acidic environment did not offer favorable surface conditions.^74^

#### 3.7.3. Effect of dose

The adsorption process is completely impacted by adsorbent dosage (Fig. S2, SI), which is investigated using doses ranged 0.002–0.02 g. The highest adsorption capacities of 94.71 mg g^−1^ were achieved using adsorbents at 0.02 g (Fig. 12b). The number of adsorbent active sites and binding sites increased, and the adsorption effectiveness and removal increased as the adsorbent dosage increased.^75^

#### 3.7.4. Effect of dye concentration and isotherm study

**Fig. 13:**
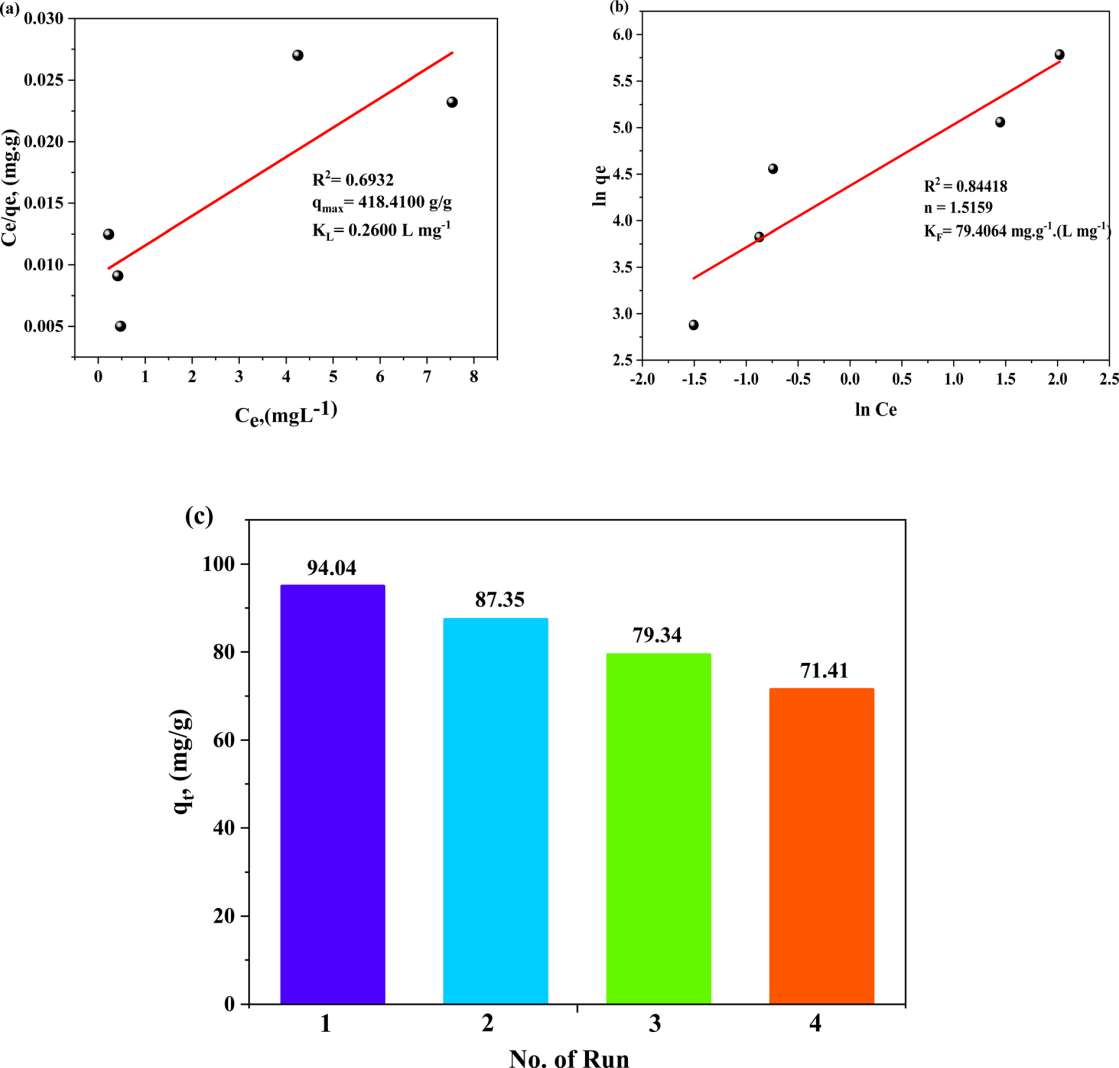
Adsorption isotherms for the adsorption of dyes on TW4. (a) Langmuir and (b) Freundlich isotherm models. (c) Reusability of TW for BF dye removal (conditions: 10 ppm dye, 0.01 g adsorbent, solution pH = 7, and 35 min of removal time).

As depicted in Fig. S3 (SI), the effect of the initial dye concentration on dye removal is discussed, showing how BF dye concentrations affect adsorption. Increasing the starting dye concentration from 2 to 40 mg L^−1^ increased the adsorption capacity of TW4 NPs from 17.78 to 324.64 mg g^−1^. Dye concentration in solution and on the material's surface varies significantly at high doses (40 mg L^−1^). This increases diffusion and produces a powerful driving force Fig. 12c,^76^ a larger concentration gradient and a stronger driving force for BF dye's adsorption at higher initial concentrations, providing more chances for dye molecules to occupy active sites and thus higher adsorption capacity.^77–79^ The most popular Langmuir and Freundlich models were utilized to study the equilibrium data of TW4 to determine which model would be best for the adsorption process design and optimization. The investigational adsorption data acquired here are well matched to the Freundlich isotherm model with a greater correlation coefficient (*R*^2^ = 0.8441) than that corresponding to the Langmuir isotherm model (*R*^2^ = 0.6832), as shown in Fig. 13(a) and (b), despite predicting a high maximum adsorption capacity (*q*_max_ = 418 g g^−1^) and Langmuir constant (*K*_L_ = 0.26 L mg^−1^). This deviation suggests that the adsorption process does not follow the assumptions of a homogeneous surface and monolayer coverage.^80,81^ In contrast, the Freundlich isotherm model indicates that adsorption occurs on a heterogeneous surface with varying energy sites and multi-layer adsorption,^82^ resulting in monomolecular coverage of the material surface during BF adsorption. The value of *n* is a kind of isotherm; for example, if 1/*n* equals zero, 0 < 1/*n* < 1, or 1/*n* > 1.61, the isotherm is unfavorable, favorable, or irreversible, respectively. The current analyzed data *n* value for TW4 is 1.510, indicating favorable adsorption.^83^ Slight surface changes introduced by doping greatly enhance surface reactivity and binding stability across a broad range of environmental factors although they may not always boost adsorption under ideal conditions. This finding has been confirmed by EIS analysis, which shows that the doped sample's effective capacitance and charge-transfer resistance, suggesting both improved surface contact stability and retained intrinsic activity.

### 3.8. Regeneration and reusability

An essential characteristic of dye adsorption is the cyclic regeneration and reutilization of TW NPs as adsorbents. After desorption, the dye was regenerated three times using methanol and DI water, and it was then dried for 24 h at 90 °C. This procedure is reversible. Under ideal adsorption conditions (pH 7 and initial BF concentration of 10 ppm under ambient conditions), the adsorbent's recovery ability was examined. Even after four adsorption cycles, the BF dye removal effectiveness was reduced from 94.0 to 71.4 mg g^−1^ (Fig. 13c). Overall, the adsorption processes between BF and TW NPs may also be impacted by the eventual loss of adsorbent mass and the dissolution of TW.^84,85^

### 3.9. Comparison of TW with other catalysts

The catalytic performance of MgO nanocatalysts was compared with that of numerous previously reported catalysts (Table 6). For example, several catalysts require high catalyst loadings, such as MgO NPs (0.05 g) and MgO@*Sargassum hystrix* (2 g). However, some of them require high dye concentrations, such as MgO NPs@*Aspergillus japonicas* (100 ppm), MgO NPs (80 ppm), MgO NPs (75 ppm), and MgO NPs (70 ppm). Instead of high catalyst loadings and high dye concentrations, these catalysts required longer reaction times. Therefore, the results demonstrate the enhanced capacity of our catalyst for the effective removal of BF.

**Table 6:** Comparative study of MgO catalysts with previously reported results

Catalyst	Preparation method	Dye	Dose (g)	Dye conc (ppm)	Time (min)	Kinetics	Freundlich			Langmuir			Ref.
							*K*	*n*	*R* ^2^	*q*_m_	*K*_L_	*R* ^2^	
MgO NPs	Green synthesis	Acid violet	0.05 g	50 ppm	20	Pseudo-first-order	0.071	2.17	0.973	47.8	52.91	0.993	86
MgO NPs@Aj	Green synthesis	Reactive black 5	0.005 g	100 ppm	40	Pseudo-second-order	23.36	1.9	0.813	204.08	0.0861	0.9422	87
MgO NPs	Green synthesis	Nigrosine dye	0.025 g	10 ppm	50	Pseudo-second-order	11.81	−333.33	0.934	—	—	0.99	88
MgO@Sh	Sol–gel	Acid black 1	2 g L^−1^	—	150	Intra-particle diffusion kinetic	—	1.8	0.985	9.34	0.067	0.985	89
MgO NPs	Microwave-assisted combustion	Trypan blue	0.2 g	25 ppm	120	Pseudo-second-order	41.68	1.01	0.999	93.54	0.95	0.990	90
MgO NPs	Chemical precipitation method	Direct sky blue	0.05 g	70 ppm	75	Pseudo-second-order	154.88	−1.92	0.924	43.75	−0.11	0.977	91
TW4 NPs	Sol–gel auto combustion	Basic fuchsin	0.01 g	10 ppm	35	Pseudo second-order	79.40	1.51	0.844	418.41	0.26	0.683	Present study

### 3.10. Antimicrobial activity and minimum lethal concentration of TW NPs

Metal nanoparticles (ZnO, SnO_2_, WO_3_, MgO, TiO_2_, *etc.*) are used in a variety of fields, including biology, electrochemistry, environmental science, and medicine.^92–94^ They have a significant surface area to volume ratio, are optical and magnetic, and have a high surface energy when compared to their bulk counterparts.^93,95^ Magnesium oxide and tungsten oxide are two metal oxides believed to be beneficial to human health. MgO is a mineral that is applied in medicine to treat specific diseases, including heartburn and stomach pain. MgO NPs might have lengthy antimicrobial effects due to their low volatility and excellent temperature stability.^96,97^ Some well-known NPs (Ag, Cu, Ti, and others) have limited economic applicability due to their high biotoxicity. To address the toxicity issue, researchers have investigated NP oxides as a safer and more effective option. Similarly, the surface modification of NPs has been proposed to broaden their applicability while reducing side toxicity.^98,99^ Cancer treatment using currently known procedures typically leads to immunosuppression in patients, putting them at high risk for serious microbial infections. It is critical to develop a novel anticancer drug with integrated antimicrobial activity.^93^ Surface-functionalized NPs are now mostly used in many disciplines, including environmental science and medicine. Antimicrobial treatments, such as antibiotics, and surface modifications to hospital facilities and equipment have been developed to reduce the prevalence of nosocomial infections. However, antibiotic resistance has rendered most of them ineffective and unsustainable. As a result, research regarding alternative materials capable of combating pathogenic microbes has continued.^100,101^

**Fig. 14:**
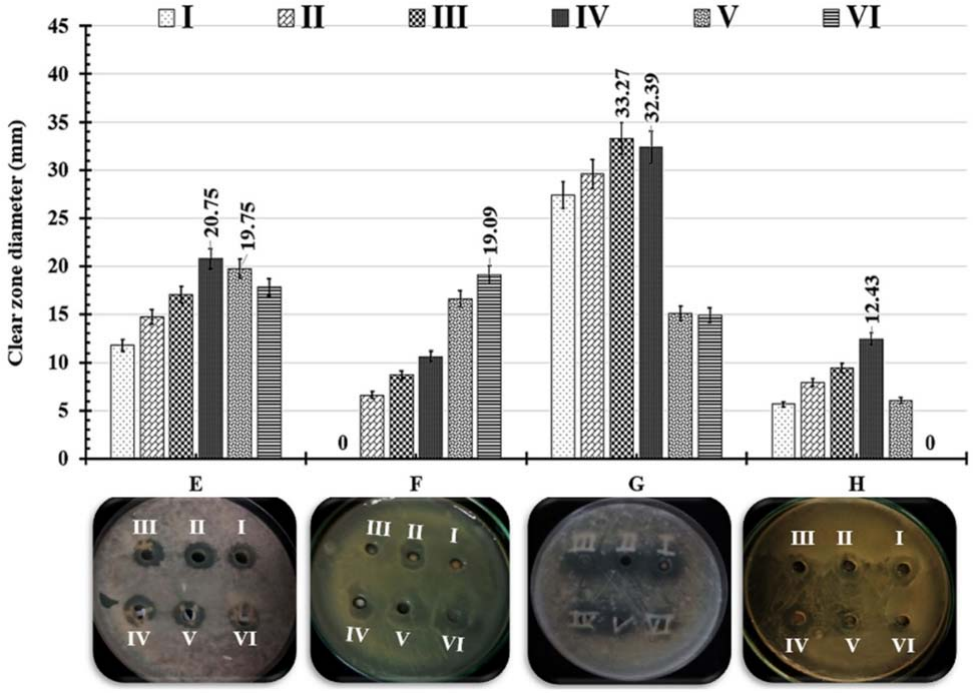
Photographs and comparative bar chart showing the inhibition zone diameter (mm) produced by different concentrations of TW NPs. (I): 10 µg mL^−1^, (II): 30 µg mL^−1^, (III): 50 µg mL^−1^, (IV): 70 µg mL^−1^, (V): 90 µg mL^−1^, and (VI): 110 µg mL^−1^ against different human pathogens.

The antimicrobial activity of prepared TW NPs was tested against Gram +ve bacteria (*Staphylococcus aureus* and *Bacillus cereus*), Gram −ve bacteria (*Klebsiella pneumoniae*, *E. coli*, *Klebsiella rhinoscleromatis*, and *Salmonella* organisms), and fungal cells, such as *Candida glabrata* and *Candida albicans*. Table 7 lists the diameters of clear zones as well as the calculated MIC for TW NPs, ranging from 10 to 110 µg mL^−1^ against all tested human pathogens. In the current investigation, Gram −ve bacteria such as *Salmonella* organisms (33.27 ± 6.49), *Klebsiella pneumoniae* (20.75 ± 1.38), and *Klebsiella rhinoscleromatis* (19.09 ± 1.45) were shown to be the most sensitive bacteria to TW NP doses at 50, 70, and 110 µg mL^−1^, respectively (Fig. 14). Furthermore, the lowest clear zone was measured as 12.43 ± 0.43 against *Escherichia coli* at 70 µg mL^−1^ of TW NPs. Moreover, Gram +ve and fungal plates did not have any distinguishable clear zones against all tested TW NP doses (Table 7). Gram-positive bacteria have thick peptidoglycan layers on their cell walls, while Gram-negative bacteria have a thin peptidoglycan layer with lipopolysaccharides on their cell walls. Based on mechanisms commonly reported for similar nanomaterials in the literature, we speculate that TW NPs may be attracted to the negative charge of lipopolysaccharides, which then would cause aggregation on the bacterial cell membrane, leading to a reduction in selective permeability. The current study does not directly confirm this mechanism; it is presented as a reasonable hypothesis assumed from earlier studies. Furthermore, based on previous studies, we suggest that these TW NPs can disrupt the communication mechanisms of pathogens, such as quorum sensing, interrupting physiological activities, and a variety of microbial behavioral patterns.^93,102^

**Table 7:** Antimicrobial efficacy of different concentrations of the TW NPs against different human pathogens by calculating the diameter of clear zones (mm ± SD)

Human pathogens	Clear zone diameters (mm ± SD) of TW NPs					
	10 µg mL^−1^	30 µg mL^−1^	50 µg mL^−1^	70 µg mL^−1^	90 µg mL^−1^	110 µg mL^−1^
*Staphylococcus aureus*	00 ± 00	00 ± 00	00 ± 00	00 ± 00	00 ± 00	00 ± 00
*Bacillus cereus*	00 ± 00	00 ± 00	00 ± 00	00 ± 00	00 ± 00	00 ± 00
*Candida glabrata*	00 ± 00	00 ± 00	00 ± 00	00 ± 00	00 ± 00	00 ± 00
*Candida albicans*	00 ± 00	00 ± 00	00 ± 00	00 ± 00	00 ± 00	00 ± 00
*Klebsiella pneumoniae*	11.73 ± 0.71	14.73 ± 2.29	17.06 ± 2.24	20.75 ± 1.38	19.75 ± 3.4	17.8 ± 1.35
*Klebsiella rhinoscleromatis*	00 ± 00	6.61 ± 0.36	8.7 ± 0.96	10.62 ± 1.23	16.61 ± 0.71	19.09 ± 1.45
*Salmonella* organisms	29.59 ± 3.15	27.42 ± 1.96	33.27 ± 6.49	23.39 ± 5.39	15.09 ± 2.27	14.89 ± 2.4
*Escherichia coli*	5.67 ± 0.06	7.89 ± 1.09	9.42 ± 2.65	12.43 ± 0.43	6.04 ± 0.5	00 ± 00

**Fig. 15:**
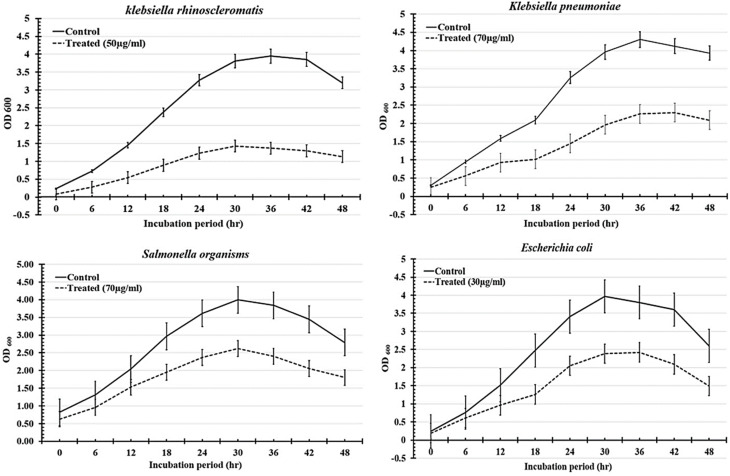
Growth curves of *Klebsiella rhinoscleromatis*, *Klebsiella pneumoniae*, *Salmonella* organisms, and *Escherichia coli* treated with different concentrations of TW NPs for 48 h compared with untreated cells (control).

**Fig. 16:**
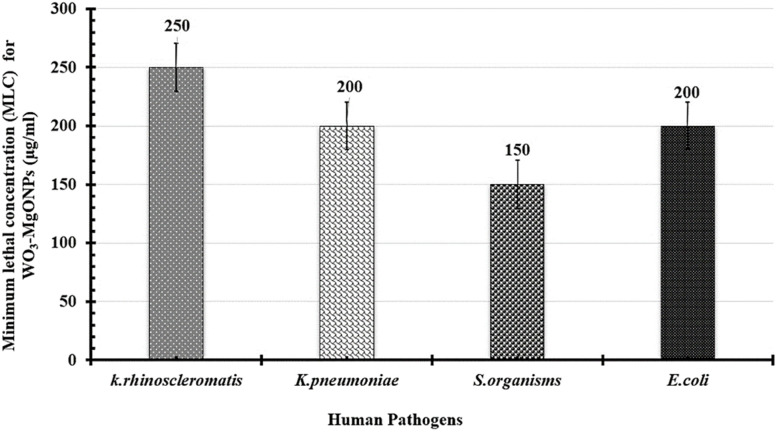
Chart showing the lowest lethal dose of TW NPs for killing 99.9% of human pathogen cells (*K. rhinoscleromatis*, *K. pneumoniae*, *S.* organisms, and *E. coli*).

The antibacterial kinetics of TW NPs in a broth culture against different Gram-negative human pathogens were also investigated by measuring OD_600_ after 48 h of growth. As shown in Fig. 15, the initial growth of all treated pathogens was a time-dependent delay compared to the corresponding control. When the concentration was within 30–70 µg mL^−1^, the TW NPs were able to severely restrict bacterial growth, which was more noticeable for *K. rhinoscleromatis* and *K. pneumoniae* than for *S.* organisms and *E. coli*. Moreover, the relative inhibitory potency (%) of WO_3_/MgONPs (µg mL^−1^) was calculated in these cases. The relative inhibitory potencies of the WO_3_/MgONPs were arranged ascendingly as follows: *K. rhinoscleromatis* (66.36%), *K. pneumoniae* (55.2%), *E. coli* (42.69%), and finally *S.* organisms (40.39%). Furthermore, the lethal dosages of TW NPs against Gram-negative bacteria were determined utilizing a dose-dependent technique (Fig. 16). These data revealed that the MLC of TW NPs was larger in *K. rhinoscleromatis* (250 µg mL^−1^) than in *K. pneumoniae* (200 µg mL^−1^) and *E. coli* (200 µg mL^−1^). However, the recorded MLC against *S.* organisms (150 µg mL^−1^) was found to be lower. The antimicrobial mechanisms of metal oxide NPs are inadequately recognized. However, oxidative stress induction, metal ion release, and non-oxidative processes are widely accepted. Based on some studies, metal oxide nanoparticles have achieved direct contact with pathogen cell membranes rather than penetrating inside them.^43,103^ For other pathogens, however, metal oxide nanoparticles penetrate their cell walls, causing intracellular disruptions, such as DNA and protein interactions.^46^ Finally, the antimicrobial mode of action was influenced not only by microbial cell structure but also by their reactivity to metal oxide nanoparticles, in addition to the shape, size, and charge of the tested metal oxide NPs.

## 4. Conclusions

MgO NPs doped with different concentrations of tungsten oxide NPs were effectively fabricated using the sol–gel/auto-combustion method. FTIR examination verified the effective doping of tungsten oxide into the MgO lattice, while XRD investigation validated the cubic crystal structure of MgO. The strongest adsorption and antibacterial activity were demonstrated by TW NPs, especially those containing 10% tungsten oxide NPs coded (TW4). The results demonstrate that TWs possess pH-dependence in the uptake of BF dye, and the maximum uptake capacities of TWs were 324.64 mg g^−1^ at high doses (40 mg L^−1^). Moreover, antibacterial qualities were shown by these nanoparticles against *K. rhinoscleromatis*, *K. pneumoniae*, *S.* organisms, and *E. coli*, among other Gram-positive and Gram-negative bacteria. Tungsten oxide doping significantly improved antibacterial activity against Gram-negative bacteria, suggesting that these nanoparticles might be useful tools for both water treatment and nanobiotechnology applications. Overall, doping is an effective way to stabilize and extend this performance under different operating conditions rather than only increasing peak efficiency, even if the pure catalyst naturally has a high adsorption efficiency.

## Author contributions

Amina Toumi and Habiba A. Hossni: methodology, investigation, formal analysis, data curation. Mohammad Abohassan: methodology, investigation, funding acquisition, formal analysis. Shahira H. EL-Moslamy: microbiology part. Heba Y. Zahran: writing – original draft, visualization, validation. Elbadawy A. Kamoun: writing – review and editing, writing – original draft, visualization, validation, supervision, project administration. V. Ganesh and Saleh M. Matar: software, resources, methodology, investigation. Ibrahim S. Yahia: writing – review and editing, writing – original draft, supervision, software, resources, project administration.

## Conflicts of interest

The authors declare that they have no known competing financial interests or personal relationships that could have appeared to influence the work reported in this paper.

## Supplementary Material

RA-016-D6RA02662A-s001

## Data Availability

The data sets used and/or analyzed during the current study are available from the corresponding authors upon reasonable request. Supplementary information (SI) is available. See DOI: https://doi.org/10.1039/d6ra02662a.

## References

[cit1] Alhariry J. (2025). *et al.*, Design, synthesis and physicochemical characterization of lysozyme stabilized silver nanocatalyst for degradation of hazardous dyes. J. Mol. Liq..

[cit2] Rani S., Kumar P., Kataria N. (2025). Development of chemically and green synthesized MgO nanoparticles for CR and BG dye adsorption from single and binary aqueous system. J. Taiwan Inst. Chem. Eng..

[cit3] Ben Aissa M. A. (2022). *et al.*, Yttrium oxide-doped ZnO for effective adsorption of basic fuchsin dye: equilibrium, kinetics, and mechanism studies. Int. J. Environ. Sci. Technol..

[cit4] El Haddad M. (2016). Removal of Basic Fuchsin dye from water using mussel shell biomass waste as an adsorbent: Equilibrium, kinetics, and thermodynamics. J. Taibah Univ. Sci..

[cit5] Elamin M. R. (2024). *et al.*, Fabrication of ternary Co_3_O4@β-Bi2O3@g-C_3_N_4_ nanosorbent for efficacy Basic Fuchsin (BF) dye decontamination. Inorg. Chem. Commun..

[cit6] Jain R. (2021). *et al.*, Green synthesis of iron nanoparticles using Artocarpus heterophyllus peel extract and their application as a heterogeneous Fenton-like catalyst for the degradation of Fuchsin Basic dye. Curr. Res. Green Sustain. Chem..

[cit7] Amendola G., Di Luca M., Sgarbossa A. (2025). Natural Biomolecules and Light: Antimicrobial Photodynamic Strategies in the Fight Against Antibiotic Resistance. Int. J. Mol. Sci..

[cit8] Parvin N., Joo S. W., Mandal T. K. (2025). Nanomaterial-Based Strategies to Combat Antibiotic Resistance: Mechanisms and Applications. Antibiotics.

[cit9] Rashid R. (2021). *et al.*, A state-of-the-art review on wastewater treatment techniques: the effectiveness of adsorption method. Environ. Sci. Pollut. Res..

[cit10] Umesh A. S., Puttaiahgowda Y. M., Thottathil S. (2024). Enhanced adsorption: Reviewing the potential of reinforcing polymers and hydrogels with nanomaterials for methylene blue dye removal. Surf. Interfaces.

[cit11] Lotha T. N. (2024). *et al.*, Advancement in Sustainable Wastewater Treatment: A Multifaceted Approach to Textile Dye Removal through Physical, Biological and Chemical Techniques. ChemistrySelect.

[cit12] Yaseen D. A. , Chapter 3 – Fundamental and mechanisms of adsorption processes for dye removal, in Engineered Biocomposites for Dye Adsorption, ed. A. H. Jagaba, et al., Elsevier, 2025, pp. 23–45

[cit13] Ba Mohammed B. (2020). *et al.*, Fe-ZSM-5 zeolite for efficient removal of basic Fuchsin dye from aqueous solutions: Synthesis, characterization and adsorption process optimization using BBD-RSM modeling. J. Environ. Chem. Eng..

[cit14] Zhang Y. (2026). *et al.*, Magnetic Trinity Platform Based on Renewable Humic Acid for Closed-Loop Management of Antibiotics via Adsorption-Detection-Catalytic Degradation Synergy. ACS Sustain. Chem. Eng..

[cit15] Abdelrahman E. A. (2025). Efficient adsorption of basic fuchsin dye using thermally engineered novel smart nanocomposites. Sci. Rep..

[cit16] Pasindu V. (2025). *et al.*, Multifunctional transition metal oxide/graphene oxide nanocomposites for catalytic dye degradation, renewable energy, and energy storage applications. RSC Adv..

[cit17] Chinthala M. (2021). *et al.*, Synthesis and applications of nano-MgO and composites for medicine, energy, and environmental remediation: a review. Environ. Chem. Lett..

[cit18] Eissa D. (2022). *et al.*, Green synthesis of ZnO, MgO and SiO_2_ nanoparticles and its effect on irrigation water, soil properties, and Origanum majorana productivity. Sci. Rep..

[cit19] Mohammed Salman K., Renuka C. G. (2023). Modified sol-gel technique for the synthesis of pure MgO and ZnO nanoparticles to study structural and optical properties for optoelectronic applications. Mater. Today: Proc..

[cit20] Gatou M.-A. (2024). *et al.*, Magnesium Oxide (MgO) Nanoparticles: Synthetic Strategies and Biomedical Applications. Crystals.

[cit21] Perera H. C. S. (2024). *et al.*, Magnesium oxide (MgO) nanoadsorbents in wastewater treatment: A comprehensive review. J. Magnesium Alloys.

[cit22] Tang H. (2022). *et al.*, Radiative cooling performance and life-cycle assessment of a scalable MgO paint for building applications. J. Clean. Prod..

[cit23] Liu W.-J. (2013). *et al.*, Mesoporous Carbon Stabilized MgO Nanoparticles Synthesized by Pyrolysis of MgCl2 Preloaded Waste Biomass for Highly Efficient CO2 Capture. Environ. Sci. Technol..

[cit24] Pereira H. (2021). *et al.*, Influence of liquid media and laser energy on the production of MgO nanoparticles by laser ablation. Opt Laser. Technol..

[cit25] Gajengi A. L., Sasaki T., Bhanage B. M. (2017). Mechanistic aspects of formation of MgO nanoparticles under microwave irradiation and its catalytic application. Adv. Powder Technol..

[cit26] Cui H. (2014). *et al.*, Synthesis and characterization of mesoporous MgO by template-free hydrothermal method. Mater. Res. Bull..

[cit27] Mageshwari K., Sathyamoorthy R. (2012). Studies on Photocatalytic Performance of MgO Nanoparticles Prepared by Wet Chemical Method. Trans. Indian Inst. Met..

[cit28] Guo X. (2016). *et al.*, Synthesis and application of several sol–gel-derived materials via sol–gel process combining with other technologies: a review. J. Sol-Gel Sci. Technol..

[cit29] Zhu Y. (2024). *et al.*, Double Layer SiO_2_-Coated Water-Stable Halide Perovskite as a Promising Antimicrobial Photocatalyst under Visible Light. Nano Lett..

[cit30] Hornak J. (2021). Synthesis, Properties, and Selected Technical Applications of Magnesium Oxide Nanoparticles: A Review. Int. J. Mol. Sci..

[cit31] N Lotha T. (2024). *et al.*, Advancement in Sustainable Wastewater Treatment: A Multifaceted Approach to Textile Dye Removal through Physical, Biological and Chemical Techniques. ChemistrySelect.

[cit32] Mahmoud H. R., Ibrahim S. M., El-Molla S. A. (2016). Textile dye removal from aqueous solutions using cheap MgO nanomaterials: Adsorption kinetics, isotherm studies and thermodynamics. Adv. Powder Technol..

[cit33] Dang H. G. (2024). *et al.*, Combination of Cu and Zn on ZIF structure for efficient degradation of basic fuchsin in aqueous solution. React. Kinet. Mech. Catal..

[cit34] Ananda A. (2022). *et al.*, Green synthesis of MgO nanoparticles using Phyllanthus emblica for Evans blue degradation and antibacterial activity. Mater. Today: Proc..

[cit35] Mohamed R. M., Shawky A., Mkhalid I. A. (2017). Facile synthesis of MgO and Ni-MgO nanostructures with enhanced adsorption of methyl blue dye. J. Phys. Chem. Solids.

[cit36] Tahir H. (2024). *et al.*, Enhancement of adsorption and photocatalytic activity of MgO nanoparticles for the treatment of textile dye using ultrasound assisted process by Response Surface Methodology. Desalination Water Treat..

[cit37] Yadav P., Saini R., Bhaduri A. (2023). Facile synthesis of MgO nanoparticles for effective degradation of organic dyes. Environ. Sci. Pollut. Res..

[cit38] Cortés-Jácome M. (2007). *et al.*, Migration and oxidation of tungsten species at the origin of acidity and catalytic activity on WO_3_-ZrO_2_ catalysts. Appl. Catal., A.

[cit39] Santiago-Ramírez C. R. (2025). *et al.*, Transition Metal Oxides (WO3-ZrO2) as Promoters and Hydrogen Adsorption Modulators in Pt/WO_3_-ZrO_2_-C Electrocatalyst for the Reduction of NO_x_. Electrochem.

[cit40] Hkiri K. (2024). *et al.*, Experimental and theoretical insights into the adsorption mechanism of methylene blue on the (002) WO_3_ surface. Sci. Rep..

[cit41] Pradeev raj K. (2018). *et al.*, Influence of Mg Doping on ZnO Nanoparticles for Enhanced Photocatalytic Evaluation and Antibacterial Analysis. Nanoscale Res. Lett..

[cit42] Rashad M. (2023). *et al.*, Dual Studies of Photo Degradation and Adsorptions of Congo Red in Wastewater on Graphene–Copper Oxide Heterostructures. Materials.

[cit43] Khan A. (2021). *et al.*, Biosynthesis and antibacterial activity of MgO-NPs produced from Camellia-sinensis leaves extract. Mater. Res. Express.

[cit44] Nassar M. S. M., Hazzah W. A., Bakr W. M. K. (2019). Evaluation of antibiotic susceptibility test results: how guilty a laboratory could be?. J. Egypt. Publ. Health Assoc..

[cit45] Khalil A. T. (2018). *et al.*, Sageretia thea (Osbeck.) modulated biosynthesis of NiO nanoparticles and their in vitro pharmacognostic, antioxidant and cytotoxic potential. Artif. Cell Nanomed. Biotechnol..

[cit46] Nguyen N.-Y. T. (2018). *et al.*, Antimicrobial Activities and Mechanisms of Magnesium Oxide Nanoparticles (nMgO) against Pathogenic Bacteria, Yeasts, and
Biofilms. Sci. Rep..

[cit47] Gebreaneniya M. F., Berhe G. G., Teklu T. (2024). Synthesis, Characterization, and Photocatalytic Activity of Cu-Doped MgO Nanoparticles on Degradation of Methyl Orange (MO). Adv. Mater. Sci. Eng..

[cit48] Schiopu A.-G. (2025). *et al.*, Ovalbumin-Mediated Biogenic Synthesis of ZnO and MgO Nanostructures: A Path Toward Green Nanotechnology. Molecules.

[cit49] Obeid M. M., Edrees S. J., Shukur M. M. (2018). Synthesis and characterization of pure and cobalt doped magnesium oxide nanoparticles: Insight from experimental and theoretical investigation. Superlattices Microstruct..

[cit50] Goswami N., Jha R. K. (2025). Structural, thermal and dielectric studies of cobalt doped ZnO nanoparticles prepared by chemical precipitation method. J. Mol. Struct..

[cit51] Muhammed Shafi P., Chandra Bose A. (2015). Impact of crystalline defects and size on X-ray line broadening: A phenomenological approach for tetragonal SnO2 nanocrystals. AIP Adv..

[cit52] Mahadik N. A. (2020). *et al.*, Evolution of lattice distortions in 4H-SiC wafers with varying doping. Sci. Rep..

[cit53] Thangabalu S., Sayed M. A., Shkir M. (2025). Dual-functional Co-doped WO3 nanoparticles for enhanced photocatalytic and antibacterial activities: Influence of morphology. Ceram. Int..

[cit54] Salman K. M., Renuka C. (2023). Modified sol-gel technique for the synthesis of pure MgO and ZnO nanoparticles to study structural and optical properties for optoelectronic applications. Mater. Today: Proc..

[cit55] Seghir B. B. (2023). *et al.*, Exploring the Antibacterial Potential of Green-Synthesized MgO and ZnO Nanoparticles from Two Plant Root Extracts. Nanomaterials.

[cit56] Benisha R. (2022). *et al.*, Catharanthus roseus leaf extract mediated Ag-MgO nanocatalyst for photocatalytic degradation of Congo red dye and their antibacterial activity. J. Mol. Struct..

[cit57] Rastegarpanah A. (2019). *et al.*, Influence of group VIB metals on activity of the Ni/MgO catalysts for methane decomposition. Appl. Catal., B.

[cit58] Proniewicz E. (2024). *et al.*, Plant-assisted green synthesis of MgO nanoparticles as a sustainable material for bone regeneration: Spectroscopic properties. Int. J. Mol. Sci..

[cit59] Ogunyemi S. O. (2020). *et al.*, The bio-synthesis of three metal oxide nanoparticles (ZnO, MnO2, and MgO) and their antibacterial activity against the bacterial leaf blight pathogen. Front. Microbiol..

[cit60] Davis E., Mott N. (1970). Conduction in non-crystalline systems V. Conductivity, optical absorption and photoconductivity in amorphous semiconductors. Philos. Mag..

[cit61] Tauc J., Menth A., Wood D. (1970). Optical and magnetic investigations of the localized states in semiconducting glasses. Phys. Rev. Lett..

[cit62] Stankic S. (2006). *et al.*, Optical surface properties and morphology of MgO and CaO nanocrystals. J. Phys. Chem. B.

[cit63] Park S. (2024). *et al.*, Machine learning-based prediction of adsorption capacity of metal-doped and undoped activated carbon: Assessing the role of metal doping. Chemosphere.

[cit64] Ebrahimzadeh F., Akbari A. (2025). Investigation the adsorption mechanisms, chemical resistance and mechanical strength of the synthesized chitosan/activated carbon composite in methylene blue removal. Sci. Rep..

[cit65] Riyad Y. M. (2023). *et al.*, Surface Functionalization of Bioactive Hybrid Adsorbents for Enhanced Adsorption of Organic Dyes. Int. J. Environ. Res. Publ. Health.

[cit66] Mohammed A. M. (2024). *et al.*, Enhanced adsorption of carbon sphere by doping with titania nanotubes for crystal violet removal: isotherm, kinetics, and thermodynamic studies. RSC Adv..

[cit67] Li D. (2020). *et al.*, Effects of Particle Size on the Structure and Photocatalytic Performance by Alkali-Treated TiO(2). Nanomaterials.

[cit68] Mohtar S. S. (2021). *et al.*, Impact of Doping and Additive Applications on Photocatalyst Textural Properties in Removing Organic Pollutants: A Review. Catalysts.

[cit69] Singh I., Birajdar B. (2017). Synthesis, characterization and photocatalytic activity of mesoporous Na-doped TiO_2_ nano-powder prepared via a solvent-controlled non-aqueous sol–gel route. RSC Adv..

[cit70] Ho Y. S., McKay G. (1999). Pseudo-second order model for sorption processes. Process Biochem..

[cit71] Jovanović A. (2018). *et al.*, Tuning the electronic and chemisorption properties of hexagonal MgO nanotubes by doping – Theoretical study. Appl. Surf. Sci..

[cit72] Nolan M. (2009). Molecular Adsorption on the Doped (110) Ceria Surface. J. Phys. Chem. C.

[cit73] Cui X. (2016). *et al.*, Effect of dopant concentration on visible light driven photocatalytic activity of Sn1−xAgxS2. Dalton Trans..

[cit74] Wani A. A. (2024). *et al.*, Aspects of superior photocatalytic dye degradation and adsorption efficiency of reduced graphene oxide multiwalled carbon nanotubes with modified ZnO-Al_2_O_3_ nanocomposites. J. Environ. Chem. Eng..

[cit75] Badawi A. K., Abd Elkodous M., Ali G. A. M. (2021). Recent advances in dye and metal ion removal using efficient adsorbents and novel nano-based materials: an overview. RSC Adv..

[cit76] Al-Arjan W. S. (2022). Zinc Oxide Nanoparticles and Their Application in Adsorption of Toxic Dye from Aqueous Solution. Polymers.

[cit77] Alkhaldi H. (2024). *et al.*, Sustainable polymeric adsorbents for adsorption-based water remediation and pathogen deactivation: a review. RSC Adv..

[cit78] Farhan binti Azha S., Ahmad A. L., Ismail S. (2015). Thin coated adsorbent layer: characteristics and performance study. Desalination Water Treat..

[cit79] Khalaf-Allah A. S. A. (2025). *et al.*, Boosting adsorption capacity of methylene blue dye by multiple functional ZnO-g-C_3_N_4_/carboxymethyl chitosan/alginate –grafted polyacrylic acid composite. Sci. Rep..

[cit80] Pereira S. K. (2022). *et al.*, A simplified modeling procedure for adsorption at varying pH conditions using the modified Langmuir–Freundlich isotherm. Appl. Water Sci..

[cit81] Mozaffari Majd M. (2022). *et al.*, Adsorption isotherm models: A comprehensive and systematic review (2010−2020). Sci. Total Environ..

[cit82] Hu J. (2010). *et al.*, Adsorption Properties of MgO(111) Nanoplates for the Dye Pollutants from Wastewater. J. Chem. Eng. Data.

[cit83] Adam F. (2022). *et al.*, Enhanced adsorptive removal of indigo carmine dye by bismuth oxide doped MgO based adsorbents from aqueous solution: equilibrium, kinetic and computational studies. RSC Adv..

[cit84] Oyewo O. A. (2020). *et al.*, Sawdust-based cellulose nanocrystals incorporated with ZnO nanoparticles as efficient adsorption media in the removal of methylene blue dye. ACS Omega.

[cit85] Duojie Z. (2024). *et al.*, Tailoring morphology of MgO with Mg-MOF for the enhanced adsorption of Congo red. ACS Omega.

[cit86] Palani Singaram G. M., Selvaraj R. (2024). Photocatalytic degradation of acid violet dye by sunlight exposure using green synthesized magnesium oxide nanoparticles. Chem. Phys. Impact.

[cit87] El-Sharkawy R. M. (2025). *et al.*, Endophytic Aspergillus japonicus mediated biosynthesises of magnesium oxide nanoparticles: sustainable dye removal and in silico molecular docking evaluation of their enhanced antibacterial activity. Microb. Cell Factories.

[cit88] Kurhade P. I. (2025). *et al.*, Optimization of photocatalytic removal of nigrosine dye using green synthesized MgO nanoparticles. Biomass Convers. Biorefinery.

[cit89] Baghban N. (2025). *et al.*, Green synthesis of magnesium oxide nanoparticles using extracts of two brown seaweeds for removal of acid black 1 dye from aqueous environments. Chem. Eng. Commun..

[cit90] Priyadarshini B., Patra T., Sahoo T. R. (2021). An efficient and comparative adsorption of Congo red and Trypan blue dyes on MgO nanoparticles: Kinetics, thermodynamics and isotherm studies. J. Magnesium Alloys.

[cit91] Noreen S. (2020). *et al.*, ZnO, MgO and FeO adsorption efficiencies for direct sky Blue dye: equilibrium, kinetics and thermodynamics studies. J. Mater. Res. Technol..

[cit92] Abd-Elhamid A. (2021). *et al.*, Synthesis, Characterization, scaling up and use of Calcium Carbonate Nanoparticles to prepare nanoporous PVC film. Egypt. J. Chem..

[cit93] Duan G. (2019). *et al.*, Robust Antibacterial Activity of Tungsten Oxide (WO_3-x_) Nanodots. Chem. Res. Toxicol..

[cit94] Naguib G. H. (2021). *et al.*, In vitro Investigation of the Antimicrobial Activity of Mouth Washes Incorporating Zein-Coated Magnesium Oxide Nanoparticles. Clin. Cosmet. Invest. Dent..

[cit95] Abbas S. (2022). *et al.*, Dual-Functional Green Facile CuO/MgO Nanosheets Composite as an Efficient Antimicrobial Agent and Photocatalyst. Arabian J. Sci. Eng..

[cit96] Ogunyemi S. O. (2019). *et al.*, Biosynthesis and characterization of magnesium oxide and manganese dioxide nanoparticles using Matricaria chamomilla L. extract and its inhibitory effect on Acidovorax oryzae strain RS-2. Artif. Cell Nanomed. Biotechnol..

[cit97] Saied E. (2021). *et al.*, The Catalytic Activity of Biosynthesized Magnesium Oxide Nanoparticles (MgO-NPs) for Inhibiting the Growth of Pathogenic Microbes, Tanning Effluent Treatment, and Chromium Ion Removal. Catalysts.

[cit98] Adabi M. (2017). *et al.*, Biocompatibility and nanostructured materials: applications in nanomedicine. Artif. Cell Nanomed. Biotechnol..

[cit99] Asad Ullah A., Munir H., Shahid M. (2019). Synthesis of Bombax malabaricum gum based silver and zinc nanoparticles and their application in controlled drug delivery. Mater. Res. Express.

[cit100] Sidhu P. K., Nehra K. (2021). Purification and characterization of bacteriocin Bac23 extracted from Lactobacillus plantarum PKLP5 and its interaction with silver nanoparticles for enhanced antimicrobial spectrum against food-borne pathogens. LWT.

[cit101] Hryniewicka A. (2021). *et al.*, Dehydroepiandrosterone derived imidazolium salts and their antimicrobial efficacy. Bioorg. Chem..

[cit102] Elsayed E. M. (2020). *et al.*, Effect of the Morphology of Tungsten Oxide Embedded in Sodium Alginate/Polyvinylpyrrolidone Composite Beads on the Photocatalytic Degradation of Methylene Blue Dye Solution. Materials.

[cit103] Gudkov S. V. (2021). *et al.*, Do Iron Oxide Nanoparticles Have Significant Antibacterial Properties?. Antibiotics.

