# Supplementary material for: Dual functionality of tungsten oxide-doped magnesium oxide nanoparticles for organic dye adsorption and antimicrobial activity
Source: RSC Adv. 2026 May 26;16(31):28488–506. doi: 10.1039/d6ra02662a (PMC13213555; doi:10.1039/d6ra02662a)
Supplement: RA-016-D6RA02662A-s001 [file RA-016-D6RA02662A-s001.pdf]

Supplementary Information (SI) for RSC Advances.
This journal is © The Royal Society of Chemistry 2026

Supplementary Information

Dual-functionality of tungsten oxide doped-Magnesium oxide nanoparticles for organic dyes adsorption and antimicrobial activity

Amina Toumi[1], Habiba A. Hossni[2], Shahira H. EL-Moslamy[3], Heba Y. Zahran[4], V. Ganesh[4], Mohammad Abohassan[5], Saleh M. Matar[6], Elbadawy A. Kamoun[7*], Ibrahim S. Yahia[4*]

[1]*Department of Health and Laboratory sciences, College of Medical and Health Sciences, Liwa University, Abu Dhabi, P.O. Box 41009, United Arab Emirates.*
[2]*Nanotechnology Section, Egyptian Company for Carbon Materials, El-Sheraton/El-Nozha, Cairo 11757, Egypt.*
[3]*Bioprocess Development Department (BID), Genetic Engineering and Biotechnology Research Institute (GEBRI), City of Scientific Research and Technological Applications (SRTA-city), New Borg El-Arab City, Alexandria 21934, Egypt.*
[4]*Laboratory of Nano-Smart Materials for Science and Technology (LNSMST), Department of Physics, Faculty of Science, King Khalid University, P.O. Box 9004, Abha, Saudi Arabia.*
[5]*Department of Clinical Laboratory Sciences, College of Applied Medical Sciences, King Khalid* University, Abha, Saudi Arabia.
[6]*Department of Chemical Engineering, College of Engineering and Computer Sciences, Jazan University, Jazan, Saudi Arabia.*
[7]*Department of Chemistry, College of Science, King Faisal University, Al-Ahsa 31982, Saudi Arabia.*

**Corresponding authors: E-mail addresses: ekamoun@kfu.edu.sa, badawykamoun@yahoo.com (E.A. Kamoun) Tel: +201283320302, dr_isyahia@yahoo.com (I.S. Yahia).*

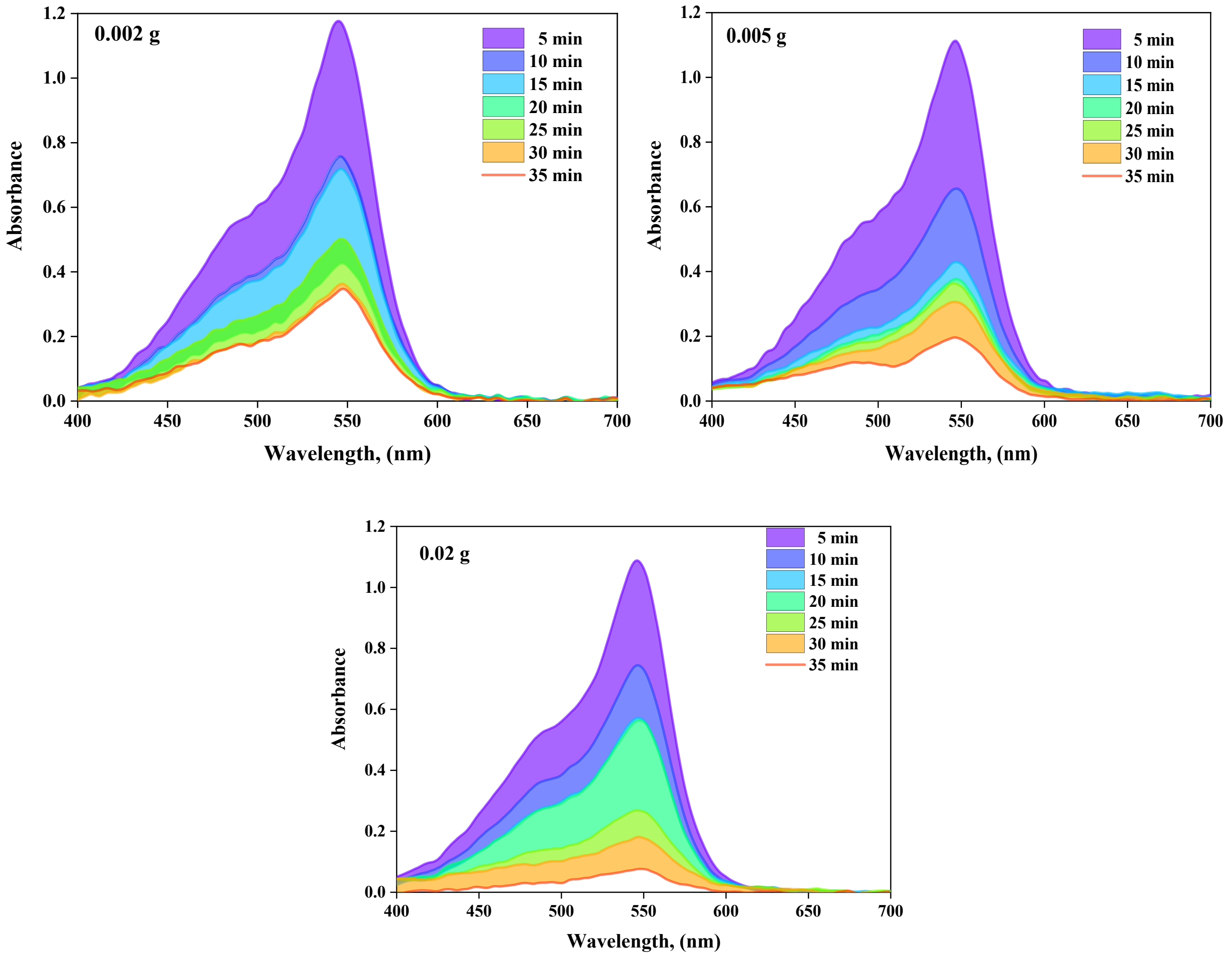

Fig. S1 UV-Vis spectra of TW4 adsorption with various doses of BF dye.

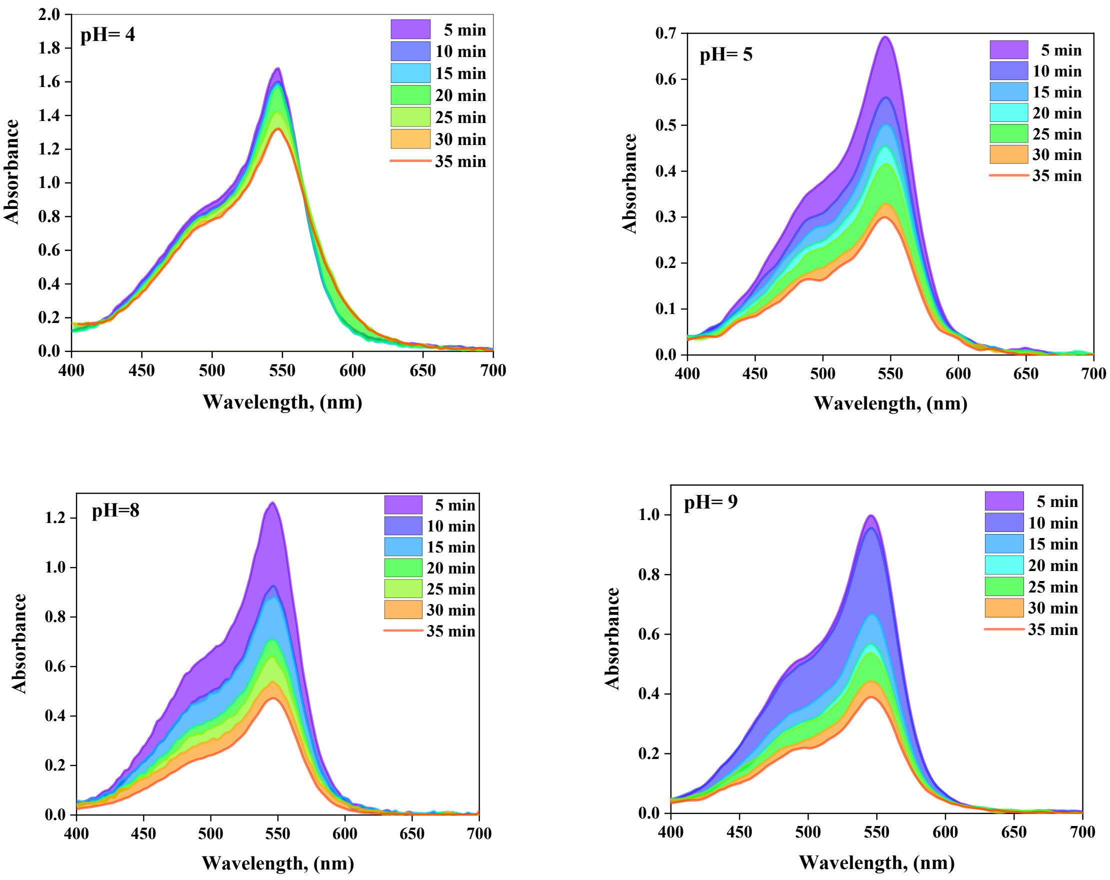

Fig. S2 UV-Vis spectra of TW4 adsorption with different pH values of BF dye.

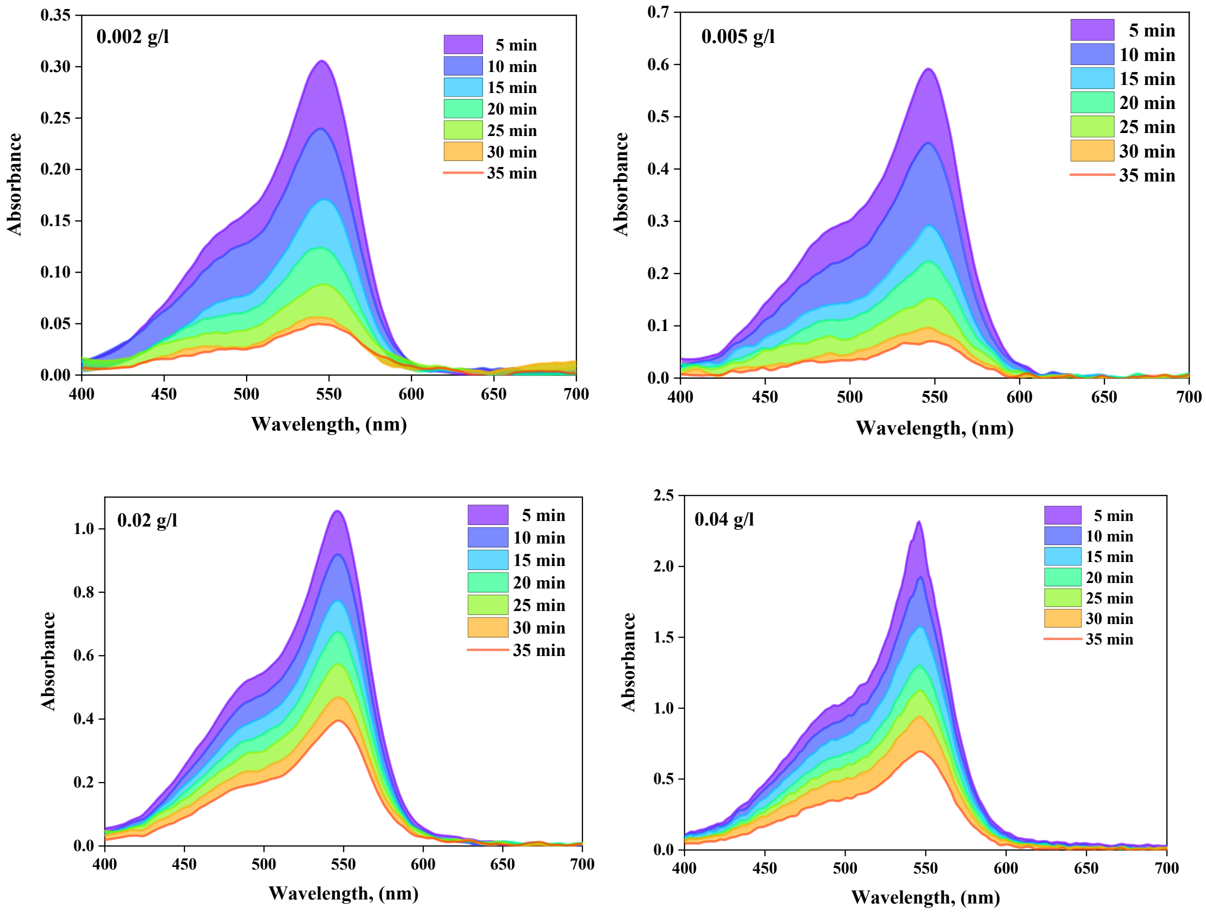

Fig. S3 UV-Vis spectra of TW4 adsorption with various dye concentrations of BF dye.
